# Defining disease stability in chronic obstructive pulmonary disease: evidence from phase 3 clinical trials

**DOI:** 10.1093/ajrccm/aamag292

**Published:** 2026-06-11

**Authors:** Dave Singh, Rianne Stacey, David M G Halpin, Surya P Bhatt, Jodie Crawford, Thomas Drury, Valentina Di Boscio, Stefanie Kolterer, MeiLan K Han

**Affiliations:** Centre for Respiratory Medicine and Allergy, Institute of Inflammation and Repair, University of Manchester, Manchester University NHS Foundation Trust, Manchester, United Kingdom; Medicines Evaluation Unit, an IQVIA business, Manchester, United Kingdom; Global Medical Affairs, General Medicines, GSK, London, United Kingdom; The College of Medicine & Health, University of Exeter Medical School, University of Exeter, Exeter, United Kingdom; Division of Pulmonary, Allergy, and Critical Care Medicine, University of Alabama at Birmingham, Birmingham, AL, United States; Development Biostatistics, GSK, London, United Kingdom; Development Biostatistics, GSK, London, United Kingdom; Global Medical Affairs, General Medicines, GSK, London, United Kingdom; Specialty Medicines, GSK, London, United Kingdom; Division of Pulmonary and Critical Care, University of Michigan, Ann Arbor, MI, United States

**Keywords:** disease stability, chronic obstructive pulmonary disease, treatment outcome, patient-reported outcome measure, prompt optimization

## Abstract

**Rationale:**

Disease stability in chronic obstructive pulmonary disease (COPD) is a low disease activity state proposed as a treatment target.

**Objectives:**

Characterize COPD stability as a composite endpoint.

**Methods:**

Post hoc analyses were carried out on IMPACT and FULFIL (fluticasone furoate/umeclidinium/vilanterol [FF/UMEC/VI] vs dual therapies) and MATINEE/METREX/METREO (mepolizumab added onto triple therapy). Stability was defined as “no moderate/severe exacerbations and no worsening from baseline in COPD Assessment Test (CAT) and FEV_1_.” We assessed whether achieving stability at week 28 was prognostic of subsequent outcomes (time to first on-treatment moderate/severe exacerbation and time to all-cause mortality [ACM]) after week 28 (IMPACT). Bayesian joint modeling evaluated the probability of achieving stability (IMPACT).

**Measurements and Main Results:**

Stability was achieved with FF/UMEC/VI by 22% of patients in IMPACT (week 52) and by 46% of patients in FULFIL (week 24); stability was achieved with mepolizumab + triple therapy by 18% of patients in MATINEE/METREX/METREO (week 52). Patients were more likely to achieve stability with triple vs dual therapy and with mepolizumab vs placebo. Many individuals achieving stability had clinically meaningful improvements in CAT and FEV_1_. Patients achieving stability at week 28 (vs patients who were not stable) had a 45.7% reduction in the risk of moderate/severe exacerbations and a 51.7% reduction in the risk of ACM after week 28. Bayesian joint modeling demonstrated a mean (SD) posterior probability of stability: 25.60% (0.66) at week 52 (FF/UMEC/VI).

**Conclusions:**

Our data provide further evidence for disease stability as an achievable COPD treatment target that predicts long-term clinical benefits in exacerbations and mortality.

At a Glance Commentary **Scientific Knowledge on the Subject:** Chronic obstructive pulmonary disease (COPD) treatment targets are needed to optimize COPD management and improve outcomes for patients. Management of COPD is moving toward proactively targeting underlying disease activity. Disease stability is recognized as a state of low disease activity. Few studies have evaluated it to date, but it is recognized as a potentially valuable treatment target that is ambitious and achievable.

**What This Study Adds to the Field:** This study further contributes to our understanding of how disease stability can be achieved across patient populations receiving different pharmacologic treatments. Importantly, it shows that achieving stability is often associated with clinically meaningful improvements in health status and lung function. It also shows that achieving disease stability over 6 months is associated with a reduction in the rate and risk of exacerbations and the risk of mortality in the subsequent 6 months. While these are post hoc analyses, they provide evidence for the potential of disease stability as a COPD treatment target to improve short- and long-term patient outcomes.

Impact on Clinical Medicine

This post hoc analysis explores and characterizes disease stability in chronic obstructive pulmonary disease (COPD), defined as no exacerbations and no worsening from baseline in COPD Assessment Test score and forced expiratory volume in 1 second over 12 months. The data show that stability is achievable to varying extents across trial populations with differing exacerbation histories and pharmacotherapy use. Achieving stability in the short term is also associated with longer-term clinical benefits on exacerbations and mortality, demonstrating its potential application as a treatment target across a wide range of patients with COPD.

## Introduction

Chronic obstructive pulmonary disease (COPD) is characterized by persistent lung inflammation that causes tissue remodeling and destruction, resulting in progressive loss of lung function.^1^ Disease activity refers to the state of the biological pathways that cause disease pathology, which can be suppressed and some pathology reversed by treatment.^1^^,^^2^ Targeting COPD disease activity can improve outcomes,^3^ whereas undertreatment and failure to suppress disease activity may result in permanent end-organ damage^4^ and contribute to the rising global disease burden.^5-7^

Low disease activity in COPD is reflected by patients not experiencing exacerbations and no worsening of other clinical features, such as symptoms.^2^ Disease stability (hereafter referred to as “stability”) is increasingly being recognized as an ambitious and achievable COPD treatment goal, and the Global Initiative for Chronic Obstructive Lung Disease (GOLD) recently defined stability as a state of low disease activity over time (ie, no exacerbations, no worsening of symptoms, and no accelerated loss of lung function).^1^ While there are few studies to date evaluating stability, preliminary evidence suggests it is achievable in the real world across a wide spectrum of patients^8^ and may serve as a meaningful marker of prognosis.^9^ Furthermore, patients recognize that achieving stability could reduce the day-to-day burden and unpredictable nature of their COPD symptoms,^10^^,^^11^ in addition to slowing down longer-term disease progression.^11-13^

We previously suggested that a COPD stability definition should encompass a composite endpoint of exacerbations, health status, and lung function.^2^ Here, we further propose that stability should be defined specifically as: no moderate/severe exacerbations and no worsening from baseline in COPD Assessment Test (CAT) score (or St George’s Respiratory Questionnaire [SGRQ]) and trough forced expiratory volume in 1 second (FEV_1_) over 12 months. To explore stability as a composite and characterize the contribution of the individual components, we conducted post hoc analyses of the pivotal Phase 3 IMPACT and FULFIL trials (in patients with symptomatic COPD at risk of exacerbations eligible for triple therapy) and a pooled population from the Phase 3 MATINEE, METREX, and METREO trials of mepolizumab (in patients on triple therapy and eligible for type 2 biologic therapy). We also investigated whether patients achieving stability experienced clinically meaningful improvements, whether achieving stability was associated with subsequent prognosis, and the effects of different pharmacologic treatments.

Some of the post hoc analyses described in this article have been previously reported in the form of abstracts at ERS 2024/2025 and ATS 2025/2026.^14-18^

## Methods

### Studies

These post hoc analyses used data from the IMPACT (NCT02164513) and FULFIL (NCT02345161) studies,^19^^,^^20^ as well as pooled data from the MATINEE (NCT04133909), METREX (NCT02105948), and METREO (NCT02105948) studies.^21^^,^^22^ Additional study details are found in the online data supplement.

### Stability with inhaled treatments

Stability was defined as a 3-component composite: no moderate/severe exacerbations and no worsening in CAT score (ie, ≥0-unit decrease) and trough FEV_1_ (ie, ≥0 mL increase) from baseline. Time points assessed included weeks 28, 52 (IMPACT), and 24 (FULFIL). Moderate exacerbations were defined as exacerbations that required treatment with oral/systemic corticosteroids and/or antibiotics (not involving hospitalization or resulting in death).^23^ Severe exacerbations were defined as exacerbations that required hospitalization or resulted in death.^19^^,^^20^ Achievement of stability at week 52 in IMPACT was also reported according to baseline blood eosinophils (<100 cells/μL, ≥100 to <300 cells/μL, ≥300 cells/μL) or smoking status at screening (current vs former). We also assessed a 2-component disease stability (DS) composite (DS-2; exacerbations and CAT components of stability only).

We further explored the individual stability components at weeks 28 and 52 (IMPACT). The threshold of no moderate/severe exacerbations was used for all analyses. Health status and lung function were evaluated using 2 thresholds: no worsening and improvement of at least the established minimal clinically important difference (MCID) from baseline; ≥2-unit decrease in CAT^24^ or a  ≥-100 mL increase in FEV_1_.^25^^,^^26^ The SGRQ total score^27^ was included as a sensitivity measure using the MCID of 4 units.^28^ For patients who did not achieve the stability definition, the magnitude of worsening for CAT and FEV_1_ was explored. This provided data on minor changes in CAT and FEV_1_ that may reflect normal variation (or age-related FEV_1_ decline). We did not include such patients in our stability definition to ensure that only individuals without clinically relevant worsening of these parameters were included.

Using IMPACT post hoc data (pooled across treatment arms), we assessed the impact of achieving stability at week 28 on subsequent outcomes after week 28 to evaluate the prognostic value of achieving stability. Outcomes included annualized rate of on-treatment moderate/severe exacerbations, time to first on-treatment moderate/severe exacerbation, and time to on- and off-treatment all-cause mortality (ACM). The contribution of the individual components of the stability composite at week 28 to these subsequent outcomes was investigated by stratifying patients according to different stability definitions with 1, 2, or 3 components. To understand the interrelatedness of the components, we stratified patients by their exacerbation status at week 52 and assessed various cutoffs for CAT (between 4-unit improvement and 4-unit worsening, in 1-unit increments) and FEV_1_ (between 100-mL improvement and 100-mL worsening, in 25-mL increments) at week 28 and 52.

#### Bayesian joint modeling

When analyzing stability at week 52 of IMPACT, the 3 components (occurrence of a moderate/severe exacerbation and no worsening from baseline in CAT and FEV_1_) were dichotomized into a single binary responder measure (responders or nonresponders). To avoid a potential reduction or loss in the detail and variability of each individual component and further validate the proposed definition of stability, we explored a Bayesian joint modeling approach using copula marginal regression^29^^,^^30^ to simultaneously model changes in CAT score, trough FEV_1_ (both normal), and the number of exacerbations (negative binomial). This approach preserves the continuous nature and distinct distributional characteristics of each endpoint and more effectively captures the correlations between endpoints.

### Stability with mepolizumab

Using pooled data from MATINEE/METREX/METREO, we sought to understand whether stability is also achievable in patients with COPD and type 2 inflammation (blood eosinophils ≥300 cells/µL) who had moderate to very severe airflow limitation and were taking triple therapy alone (placebo) or in combination with mepolizumab.^21^^,^^22^ We assessed stability at week 52 in the overall pooled population and stratified by postbronchodilator airflow limitation (GOLD grades 2-4)^1^ and chronic bronchitis status at screening (defined based on SGRQ or investigator assessed). Additional analyses evaluated stability using the SGRQ instead of CAT as a sensitivity analysis. We also assessed an adjusted composite endpoint to understand the impact of small variations in CAT: no moderate/severe exacerbations, ≤1-unit worsening in CAT, and no worsening in FEV_1_.

### Statistical analysis

We report the proportion of patients achieving each individual outcome of the stability composite by treatment arm at week 24 (FULFIL), week 28 (IMPACT), and week 52 (IMPACT and MATINEE/METREX/METREO). The likelihood of patients achieving stability at week 52 (IMPACT and MATINEE/METREX/METREO) and week 24 (FULFIL) was analyzed using a log-binomial model to estimate adjusted risk ratios (aRRs), with covariates of treatment group, sex, smoking status at screening, geographical region, baseline FEV_1_, baseline CAT score, and exacerbation history. Further statistical information can be found in the online data supplement.

Annualized on-treatment moderate/severe exacerbation rates and time to first on-treatment exacerbation were analyzed using negative binomial and Cox proportional hazards models, respectively, while times to on- and off-treatment ACM were summarized using Kaplan-Meier estimates, with hazard ratios (HRs) and 95% confidence intervals generated from a Cox proportional hazards model; full details are provided in the online data supplement. On- and off-treatment deaths were those that occurred after week 28 up to 7 days after stopping study treatment (inclusive) for patients who completed the study or through the projected week 52 date plus 7 days for patients who prematurely discontinued study treatment. Patients were included only if they were on treatment for at least 28 weeks. No imputation was performed for missing data; patients who discontinued the study prior to the time point of interest or had missing responses in any individual component were considered not to have met the composite endpoint. Sensitivity analyses were also performed to evaluate the impact of missing data on the primary DS analysis. The analysis was repeated by imputing missing data using the jump-to-reference multiple imputation method, based solely on treatment data. This was done once using fluticasone furoate/vilanterol (FF/VI) as the reference arm, and once using umeclidinium/VI (UMEC/VI) as the reference arm. Analyses are post hoc and were not adjusted for multiplicity.

In the Bayesian joint modeling approach (IMPACT), covariates included in each marginal model were treatment group, sex, smoking status at screening, geographical region, and baseline FEV_1_, baseline CAT score, or exacerbation history for each respective model. Posterior samples for the probability of achieving DS were created via G-computation^31^ and Monte Carlo integration. The results were summarized using posterior means and 95% highest posterior density credible intervals (CrI). Further details on the modeling approach can be found in the online data supplement.

All analyses were undertaken using SAS Viya 4 (SAS Institute Inc., Cary, NC, USA).

## Results

The intention-to-treat populations analyzed included 10 355 patients from IMPACT, 1810 patients from FULFIL, and 1146 patients from the pooled MATINEE/METREX/METREO eosinophilic population. The baseline disease characteristics of the studies analyzed are shown in Tables S1 and S2.

### Stability with inhaled treatments

#### Proportion of patients achieving stability

**Figure 1:**
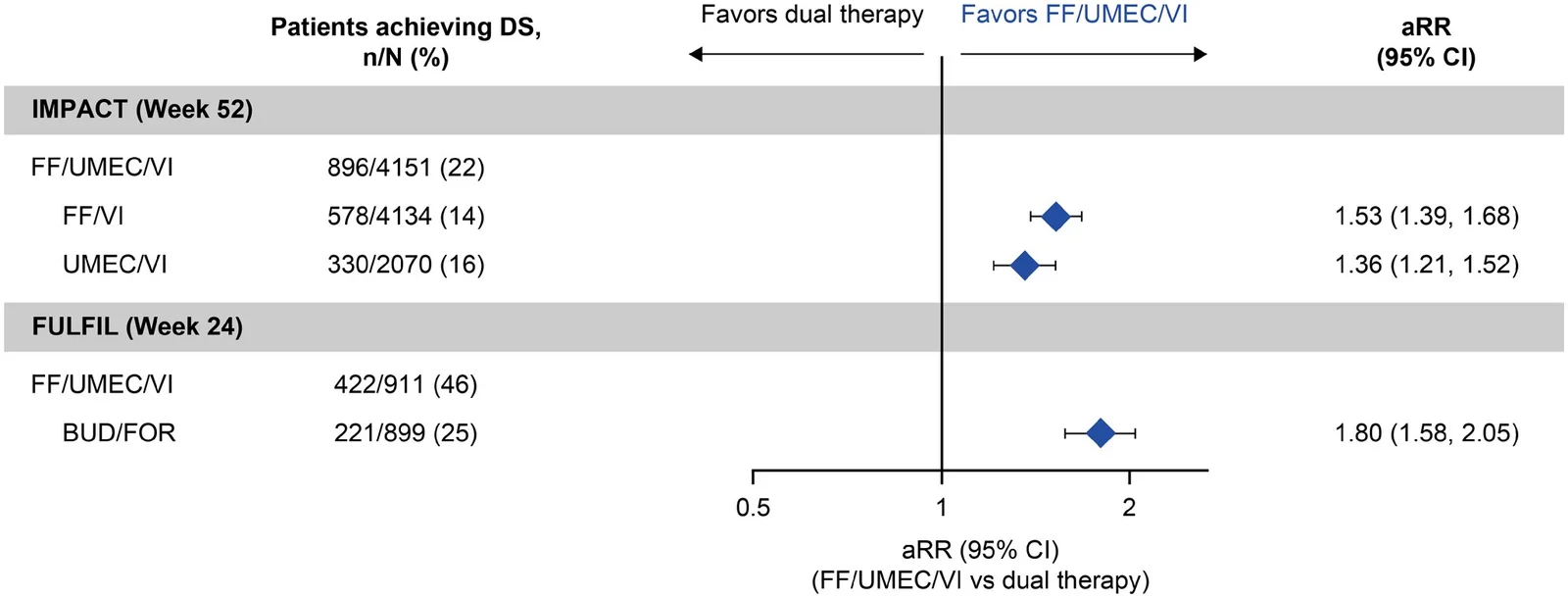
Analysis of DS status at week 52. DS is defined as no moderate/severe exacerbations and no worsening in CAT score and trough FEV_1_ from baseline. Analyzed using a log-binomial model and covariates of treatment group, sex, smoking status at screening, geographical region, baseline FEV_1_, baseline CAT score, and exacerbation history. Patients with missing CAT responses or FEV_1_ measurements were considered to have not met the DS criteria at that time point. Includes on-treatment data only. aRR, adjusted risk ratio; BUD, budesonide; CAT, COPD Assessment Test; COPD, chronic obstructive pulmonary disease; DS, disease stability; FEV_1_, forced expiratory volume in 1 second; FF, fluticasone furoate; FOR, formoterol; N, number of patients per treatment; n, number of patients achieving DS; UMEC, umeclidinium; VI, vilanterol.

At week 52 in IMPACT, 22%, 14%, and 16% of patients receiving FF/UMEC/VI, FF/VI, and UMEC/VI, respectively, achieved stability at week 52; corresponding values for FULFIL at week 24 were 46% (FF/UMEC/VI) and 25% (budesonide/formoterol]). Patients were more likely to achieve stability with FF/UMEC/VI vs FF/VI (aRR, 1.53; 95% CI, 1.39-1.68) and vs UMEC/VI (aRR, 1.36; 95% CI, 1.21-1.52) at week 52 in IMPACT and vs budesonide/formoterol at week 24 in FULFIL (aRR, 1.80; 95% CI, 1.58-2.05) (Figure 1). In the sensitivity analyses using a jump-to-reference method to impute missing data, the aRRs for FF/UMEC/VI vs FF/VI and UMEC/VI were similar to the primary analysis (Table S3), confirming its robust nature. With FF/UMEC/VI treatment, the proportion of individuals achieving stability at week 52 in IMPACT was similar, regardless of baseline blood eosinophil levels (Table S4). In contrast, the proportion of individuals treated with UMEC/VI who reached stability was lower at higher blood eosinophil counts, giving a difference between FF/UMEC/VI and UMEC/VI that varied by blood eosinophil count. Stability levels were similar in current and ex-smokers (Table S4).

**Figure 2:**
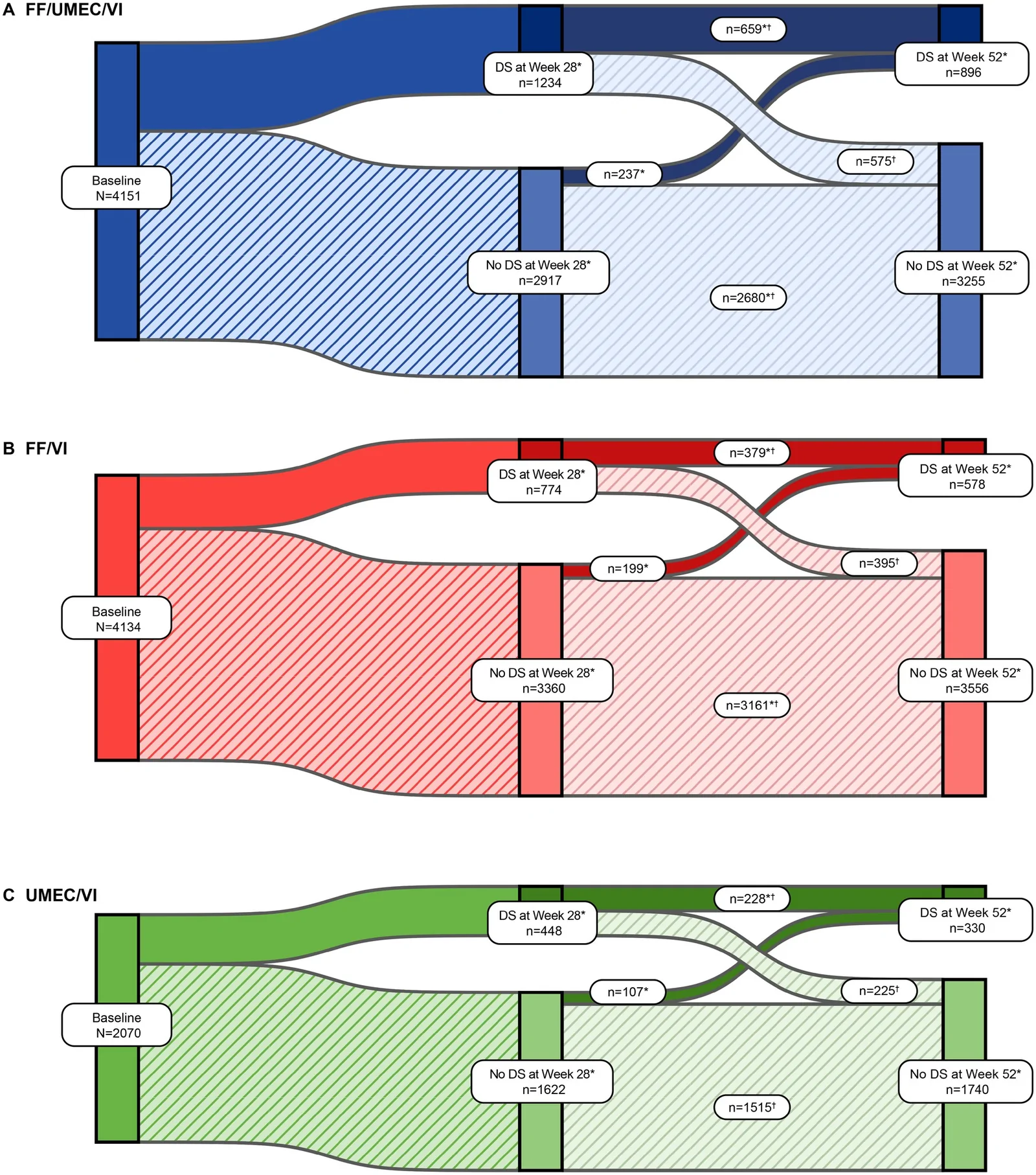
Sankey plots of DS status at week 28 and week 52 in the FF/UMEC/VI (A), FF/VI (B), and UMEC/VI (C) treatment arms. *From the original baseline (ie, start of treatment intervention). ^†^From week 28. Note: DS is defined as a 3-component composite of no moderate/severe exacerbations and no worsening in CAT score and trough FEV_1_ from baseline. Patients who discontinued the study prior to the time point of interest (week 28 and week 52 for IMPACT; week 24 for FULFIL) or had missing responses in any individual components were considered to have not met the composite endpoint. Includes on-treatment data only. CAT, COPD Assessment Test; COPD, chronic obstructive pulmonary disease; DS, disease stability; FEV_1_, forced expiratory volume in 1 second; FF, fluticasone furoate; N, number of patients in treatment group at baseline; n, number of patients achieving the composite measure; UMEC, umeclidinium; VI, vilanterol.

To illustrate the changes in stability status over time, the overall trajectories of patients from baseline to week 28 and from weeks 28 to 52 in IMPACT are depicted in Figure 2. Of the patients who were not stable at week 28 (n = 2917/4151), 8% (n = 237/2917) in the FF/UMEC/VI arm subsequently achieved stability by week 52.

**Figure 3:**
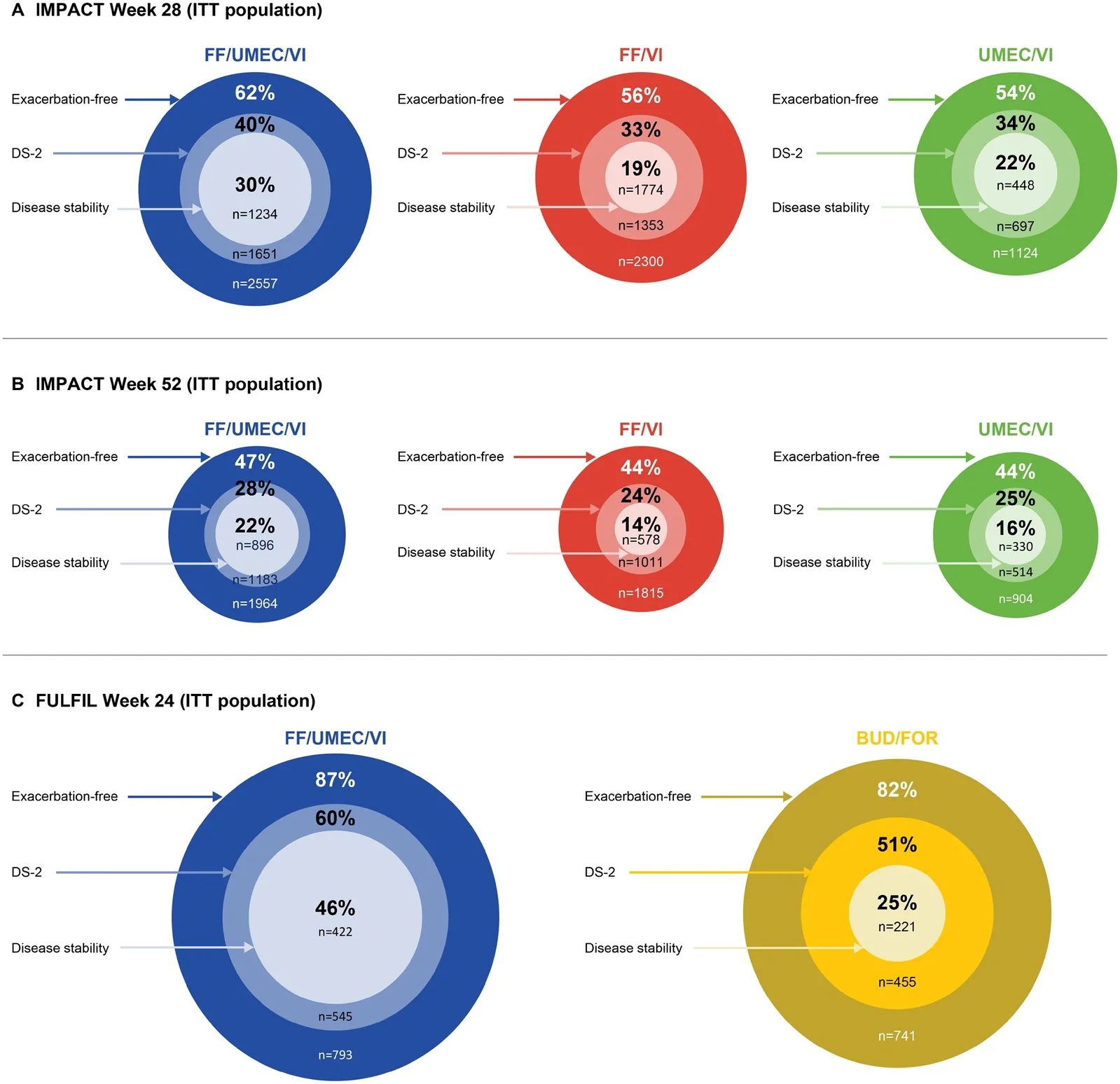
Proportion of patients who achieved DS-2 and DS in IMPACT (weeks 28 and 52) and FULFIL (week 24). Note: DS-2 is defined as a 2-component composite of no moderate/severe exacerbations and no worsening in CAT from baseline; disease stability is defined as a 3-component composite of no moderate/severe exacerbations and no worsening in CAT and FEV_1_ from baseline. Patients who discontinued the study prior to the time point of interest (week 28 and week 52 for IMPACT; week 24 for FULFIL) or had missing responses in any individual components were considered as having not met the composite endpoint. Includes on-treatment data only. BUD, budesonide; CAT, COPD Assessment Test; COPD, chronic obstructive pulmonary disease; DS, disease stability; DS-2, disease stability 2-component composite; FEV_1_, forced expiratory volume in 1 second; FF, fluticasone furoate; FOR, formoterol; ITT, intention to treat; n, number of patients achieving the measure; UMEC, umeclidinium; VI, vilanterol.

To understand stability when spirometry is not available or performed routinely, we compared DS-2 (exacerbations and CAT) vs the 3-component stability composite. More patients achieved DS-2 as fewer criteria were used. More patients also achieved stability at week 28 than at week 52 in IMPACT for both DS-2 and 3-component stability (Figure 3).

#### Improvement in patient-reported outcomes or FEV_1_

**Figure 4:**
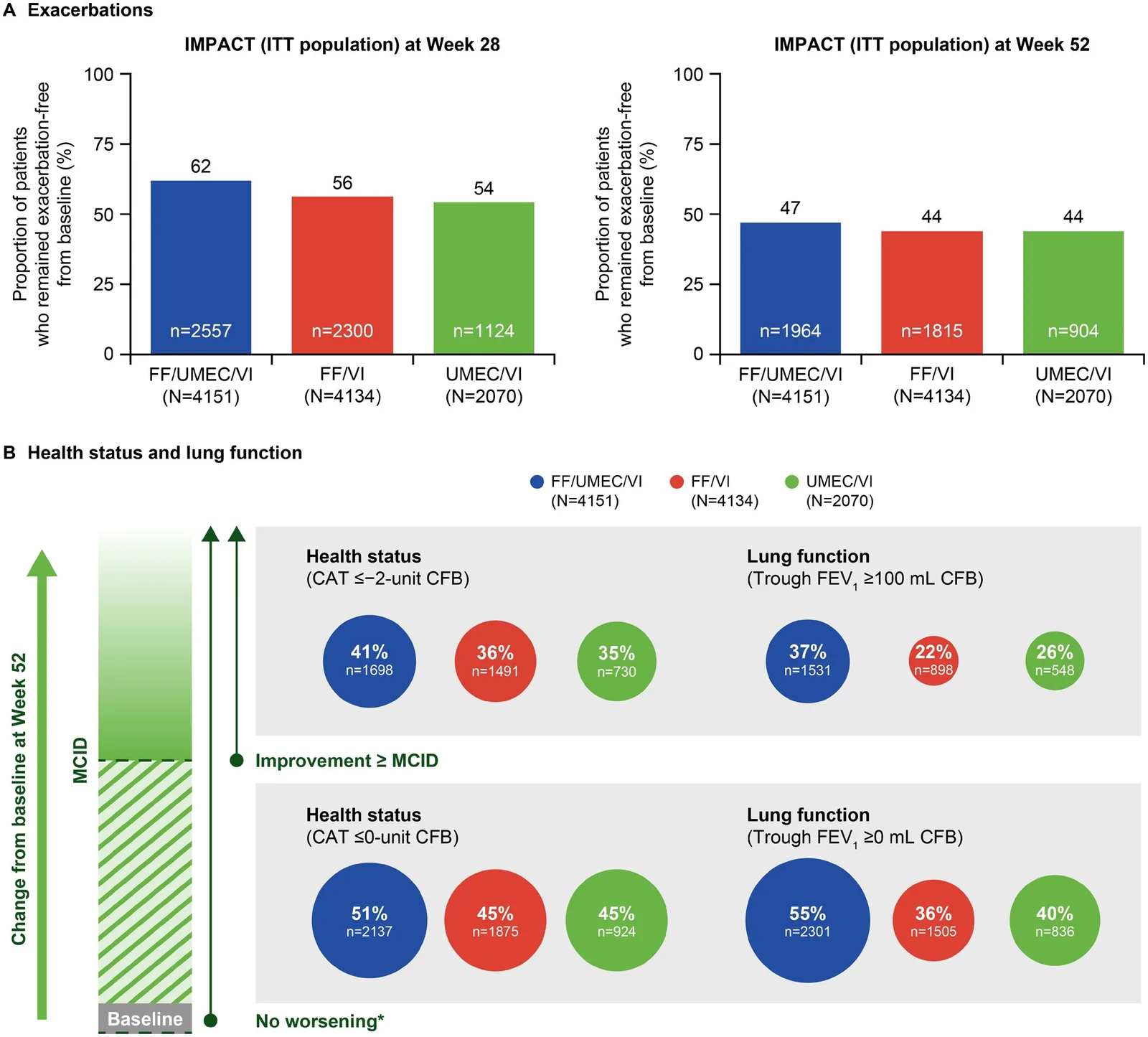
Proportion of patients who achieved the individual components of DS in IMPACT. *Patients who experienced improvements equal to or greater than the MCID were also counted within the “no worsening” threshold. Patients who discontinued study prior to the timepoint of interest were considered to have not met the “no moderate/severe exacerbations since baseline at week 52” criteria. Patients did not have a “no worsening” or “improvement” status derived if the baseline trough FEV_1_/CAT score was missing at week 52. Includes on-treatment data only. CAT, COPD Assessment Test; CFB, change from baseline; COPD, chronic obstructive pulmonary disease; DS, disease stability; FEV_1_, forced expiratory volume in 1 second; FF, fluticasone furoate; ITT, intention to treat; MCID, minimal clinically important difference; n, number of patients achieving the measure; UMEC, umeclidinium; VI, vilanterol.

In those who achieved stability in the FF/UMEC/VI arm at week 52 of IMPACT, 58% (n = 523/896) achieved clinically meaningful improvements exceeding the MCID in both CAT and FEV_1_. Additionally, most patients with no worsening from baseline in CAT or FEV_1_ individually also experienced improvements exceeding the MCIDs (week 52: 79% [n = 1698/2137] for CAT; 67% [n = 1531/2301] for FEV_1_) (Figure 4B). Similar results were observed at week 28 of IMPACT (Table S5). In the SGRQ sensitivity analysis, the results were consistent with those for CAT (Table S6).

**Figure 5:**
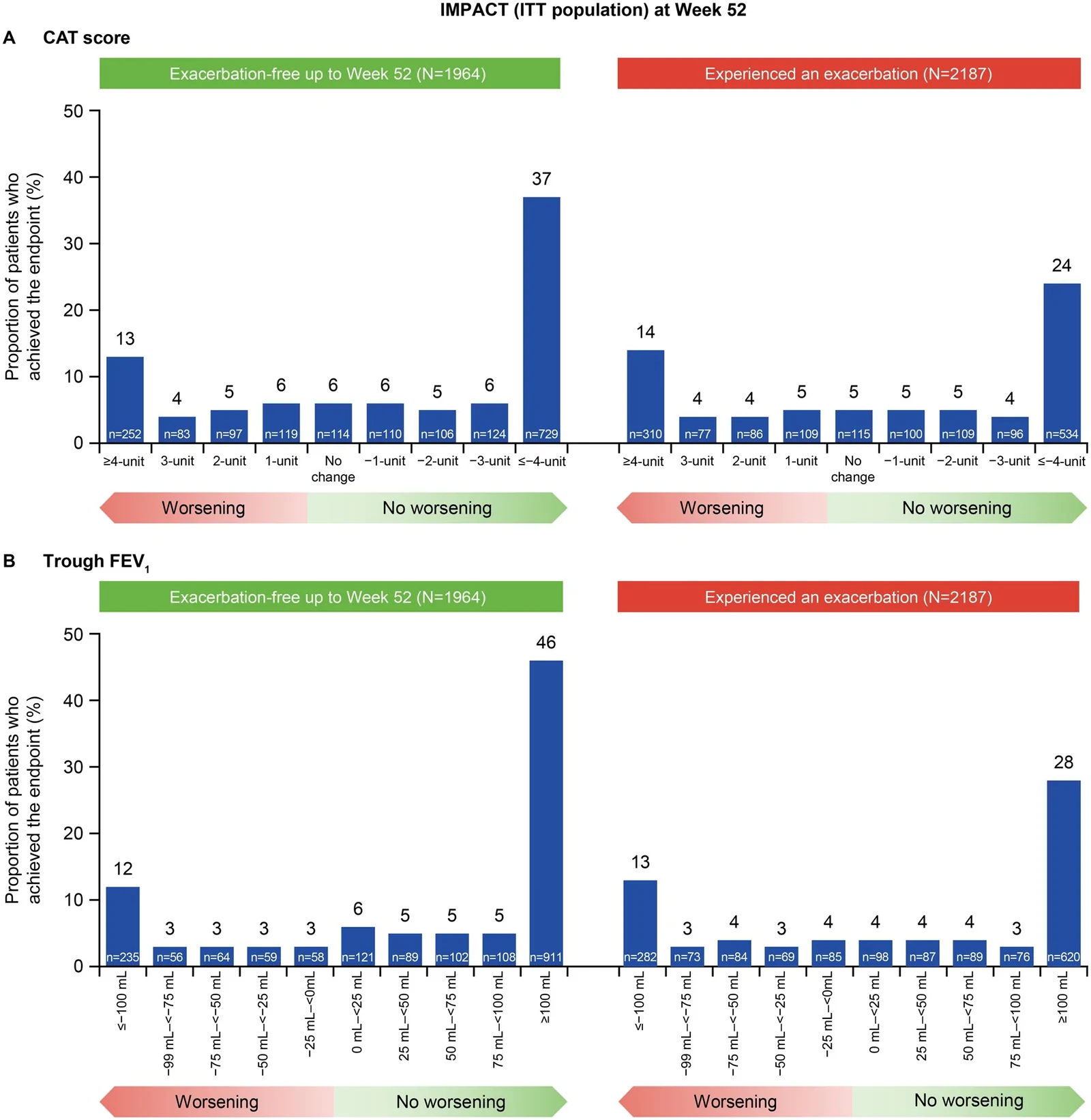
Proportion of patients in the FF/UMEC/VI arm who achieved various thresholds for CAT score and trough FEV_1_ at week 52 (IMPACT), stratified by exacerbation status at week 52. Note: patients were exacerbation-free if no moderate/severe exacerbations occurred between baseline and week 52. Patients did not have a “worsening” or “no worsening” status derived if baseline trough FEV_1_/CAT score was missing at week 52. Patients who discontinued study prior to the time point of interest were considered to have not met the “no moderate/severe exacerbations” criteria. Includes on-treatment data only. CAT, COPD Assessment Test; COPD, chronic obstructive pulmonary disease; FEV_1_, forced expiratory volume in 1 second; FF, fluticasone furoate; ITT, intention to treat; N, number of patients by exacerbation status; n, number of patients achieving the endpoint; UMEC, umeclidinium; VI, vilanterol.

For patients who were exacerbation-free up to week 52, approximately half of the FF/UMEC/VI arm achieved an improvement from baseline in CAT (49%; n = 959/1964) or FEV_1_ (46%; n = 911/1964) of at least the MCID at week 52 (Figure 5), with a similar pattern observed at week 28 (Table S7). Data for the dual-therapy treatment arms showed similar patterns (Figure 4; Tables S5, S6, and S7).

The range of changes in CAT and FEV_1_ at week 52 stratified by exacerbation status is shown in Figure 5. Only a small proportion of exacerbation-free patients did not meet the stability criteria due to small changes in these parameters, such as a 1-point increase in CAT (6%). Most failures to achieve stability were due to a relatively large worsening of CAT (≥2 points) or FEV_1_ (≥100 mL).

#### Prognostic value of the stability composite

We assessed how stability status at week 28 was associated with key clinical outcomes at week 52 (irrespective of treatment and regardless of stability status at week 52). Patients who achieved stability at week 28 experienced a 47% reduction in the annualized rate of moderate/severe exacerbations (rate ratio, 0.53; 95% CI, 0.48-0.59) compared with those who did not and a 45.7% reduction in the risk of moderate/severe exacerbations (HR, 0.54; 95% CI, 0.49-0.60) after week 28 (Table S8). The reduction in the annualized rate of moderate/severe exacerbations was greater when stability was not met due to an exacerbation occurring after week 28 (62%; including those who also did not meet the CAT/FEV_1_ criteria) than when it failed for reasons other than exacerbations (14%; Table S9). A similar result was observed for the reduction in risk of exacerbation after week 28 when assessing the reasons for not meeting the stability components (Table S8).

**Figure 6:**
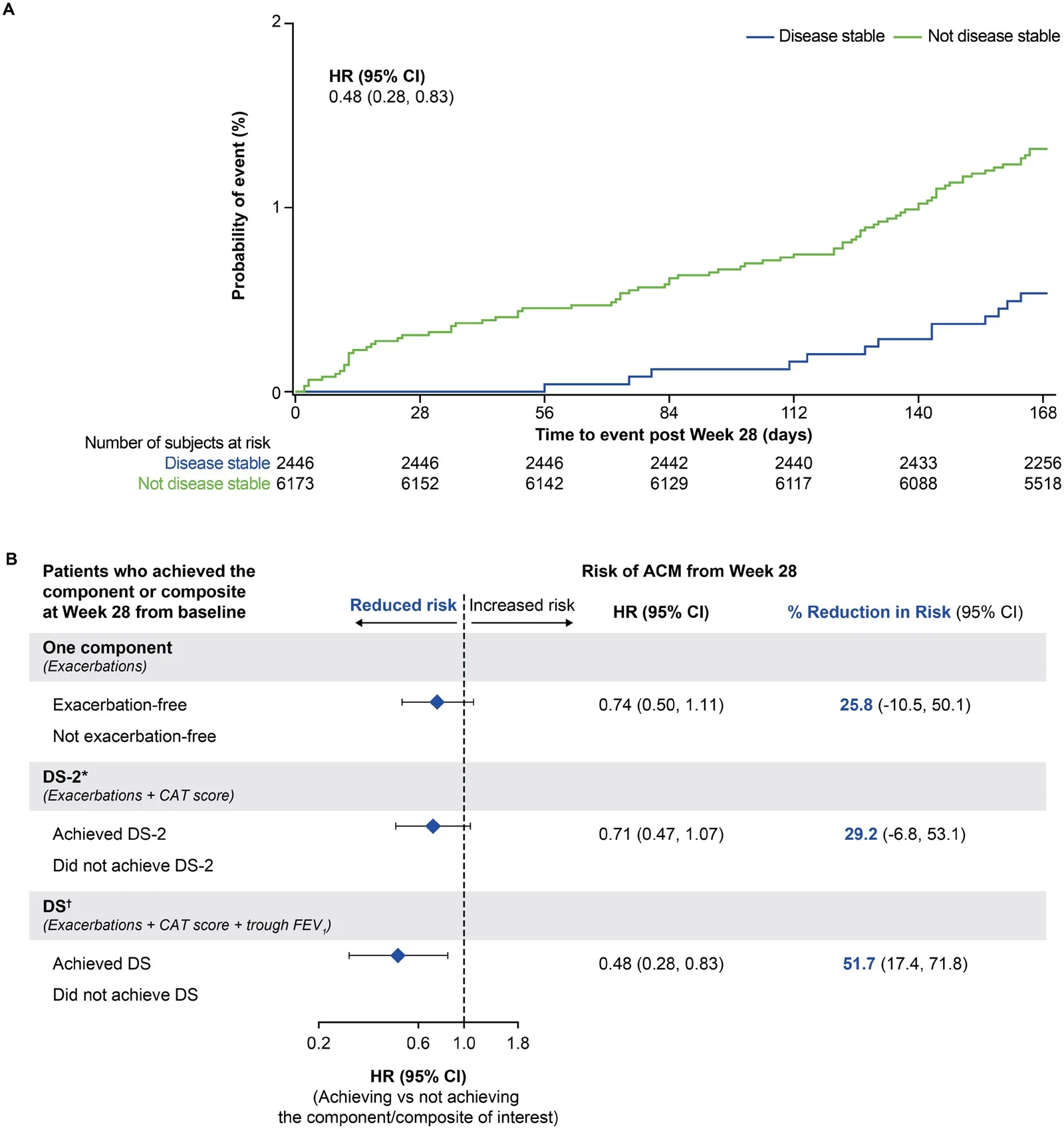
(A) Kaplan-Meier plot of time to ACM after week 28 (on- and off-treatment with Additional Vital Status follow-up) by stability status at week 28 (B) and analysis of the impact of achieving 1, 2, or 3 stability components at week 28 on risk of ACM from after week 28. Note: hazard ratio and 95% confidence interval are from a Cox proportional hazards model with covariates of treatment group, sex, age, and disease stability status at week 28. Percentage reduction in risk is calculated as (1 – hazard ratio) * 100. Patients are included only if they are on treatment for at least 28 weeks. On- and off-treatment deaths are those that occur after week 28 date and up to and including the latest of (study treatment stop date and projected week 52 date [based on treatment start date]) + 7 days. *DS-2 is defined as a 2-component composite of no moderate/severe exacerbations and no worsening in CAT from baseline. ^†^DS is defined as a 3-component composite of no moderate/severe exacerbations and no worsening in CAT and FEV_1_ from baseline. ACM, all-cause mortality; CAT, COPD Assessment Test; COPD, chronic obstructive pulmonary disease; DS, disease stability; DS-2, disease stability 2-component composite; FEV_1_, forced expiratory volume in 1 second; HR, hazard ratio.

There was a 51.7% reduction in the risk of ACM after week 28 if stability was achieved by week 28 (HR, 0.48; 95% CI, 0.28-0.83; *P* < .01; Figure 6A; Table S8). The reduction in ACM risk was 43.0% when stability was not met due to an exacerbation and 57.7% when it failed for reasons other than exacerbations (Figure S1).

To further examine the contribution of the individual components, we evaluated 1-, 2-, or 3-component definitions of stability at week 28 on the subsequent risk of ACM after week 28 (compared with those who did not achieve the component/composite). Using a 1-component exacerbation-free definition, there was a 25.8% reduction in the risk of ACM, which rose to 29.2% for patients achieving DS-2 (exacerbations and CAT) and further increased to 51.7% for patients who achieved the 3-component stability composite (Figure 6B), underscoring the additive value of all 3 stability components.

#### Bayesian joint modeling

**Figure 7:**
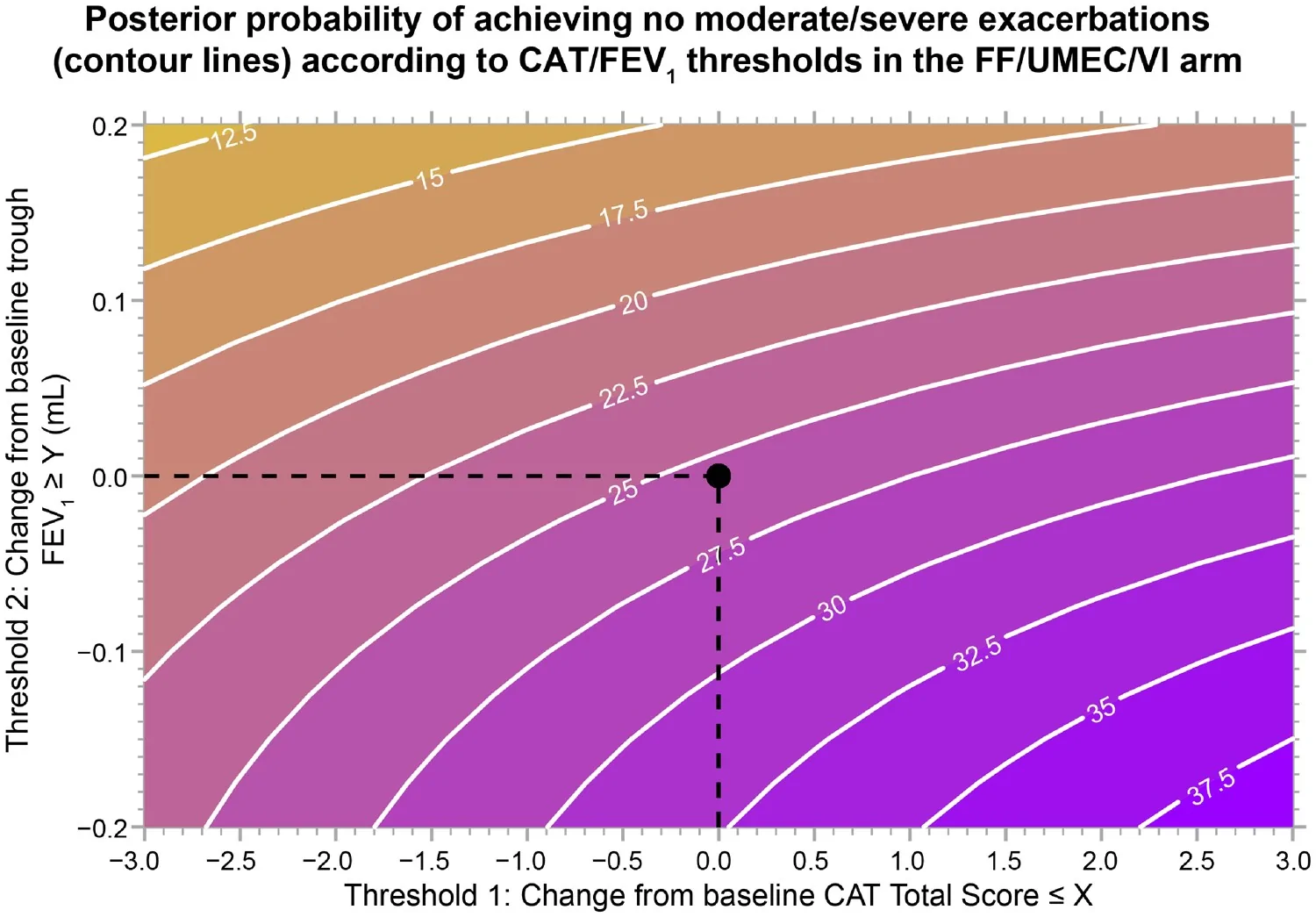
Posterior probability of patients achieving no moderate/severe exacerbations, according to CAT and trough FEV_1_ thresholds using Bayesian joint modeling via copula marginal regression (FF/UMEC/VI arm). Positive CAT values indicate greater symptom improvement, whereas negative trough FEV_1_ values indicate greater improvement in lung function. DS at week 52 was defined as no moderate/severe exacerbations since baseline, change from baseline in CAT total score of ≤X, and change from baseline in trough FEV_1_ ≥ Y (mL). Posterior probability of DS was estimated jointly using Bayesian Gauss copula marginal regression. All marginal models included terms for treatment, smoking status at screening, and geographical region; the marginal models for change from baseline in trough FEV_1_ and CAT total score included corresponding terms for baseline; the marginal exacerbation model also included terms for sex and exacerbation history. The contour surface plot shows the posterior probability of achieving no moderate/severe exacerbations along with achieving combinations of change from baseline in CAT total score (x-axis) and change from baseline in trough FEV_1_ (y-axis). As an example, the posterior probability of achieving no exacerbations, with at least a zero change from baseline in CAT score and at least a zero change in trough FEV_1_, would be the contour values at the point [0,0] on the plot. CAT, COPD Assessment Test; COPD, chronic obstructive pulmonary disease; DS, disease stability; FEV_1_, forced expiratory volume in 1 second; FF, fluticasone furoate; UMEC, umeclidinium; VI, vilanterol.

Using the stability thresholds in IMPACT, the mean (SD) posterior probability of stability was 25.60% (0.66) (95% highest posterior density [HPD] CrI, 24.35-26.93) for FF/UMEC/VI, 19.33% (0.61) (95% HPD CrI, 18.15-20.55) for FF/VI, and 22.13% (0.85) (95% HPD CrI, 20.58-23.78) for UMEC/VI (Figure 7; Figure S2), consistent with the values obtained in the main analysis of stability in IMPACT. Small changes in CAT/FEV_1_ thresholds had a limited impact on the overall posterior probability of achieving stability; for example, allowing a 1-unit worsening in CAT increased the posterior probability from 25.60% to approximately 27.50% for FF/UMEC/VI.

### Stability with mepolizumab

**Figure 8:**
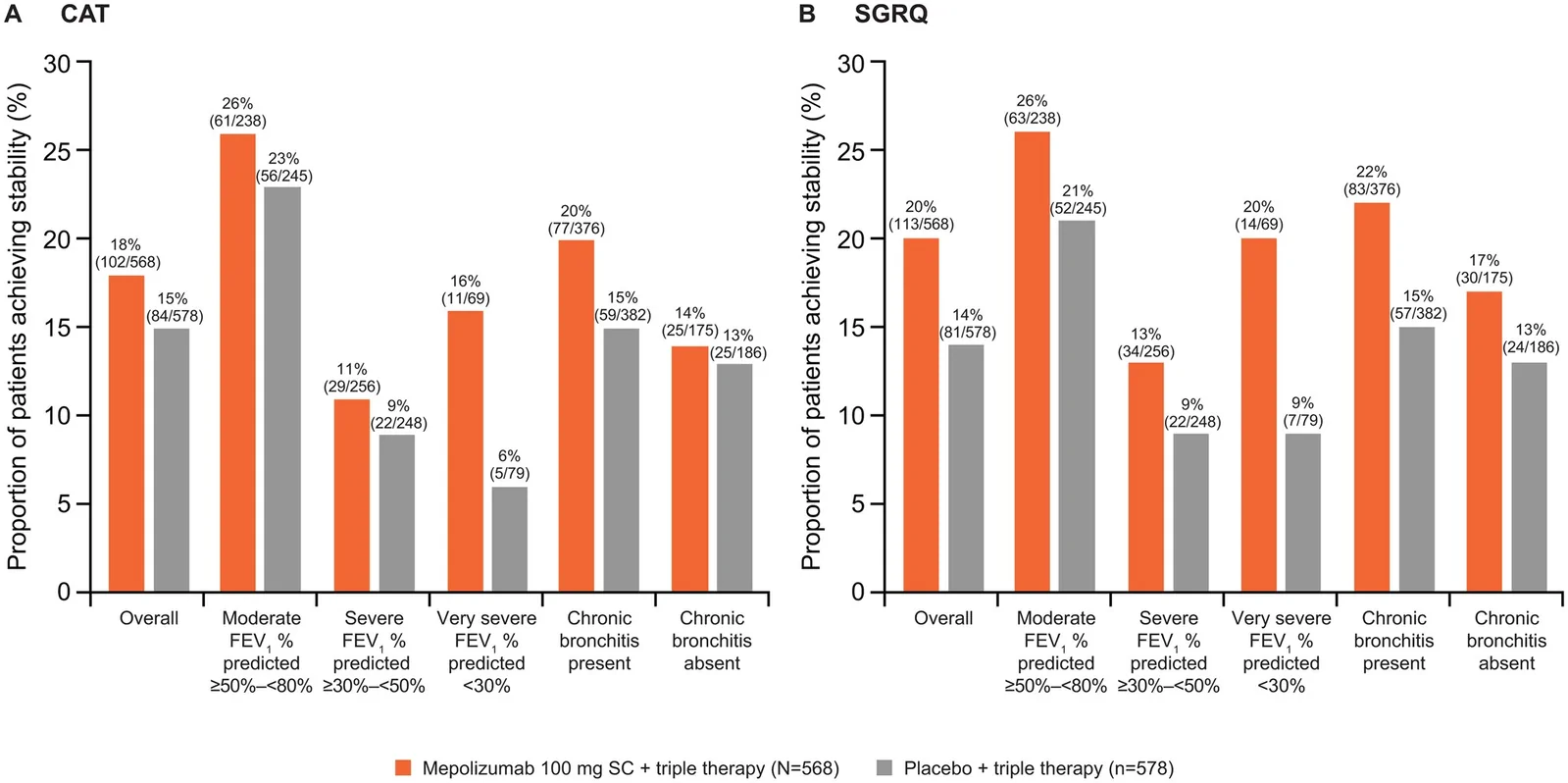
Proportion of patients in MATINEE, METREX, and METREO who achieved disease stability overall, by the degree of airflow limitation and by presence of chronic bronchitis, using the (A) CAT or (B) SGRQ stability definitions. Disease stability was defined as no moderate/severe exacerbations and no worsening in CAT (or SGRQ) and FEV_1_ since baseline. Note: patients who discontinued the study prior to the time point of interest (week 52) or had missing responses in any individual components were considered to have not met the composite endpoint. Mepolizumab 300 mg SC treatment was included in the model but is not presented here. CAT, COPD Assessment Test; COPD, chronic obstructive pulmonary disease; FEV_1_, forced expiratory volume in 1 second; SC, subcutaneous; SGRQ, St George’s Respiratory Questionnaire.

There were 568 patients included in the mepolizumab + triple therapy arm (MT) and 578 patients in the placebo + triple therapy arm (PT) with blood eosinophils ≥300 cells/μL across all 3 Phase 3 trials. Overall, 18% (n = 102/568) of MT patients achieved stability vs 15% (n = 84/578) of PT patients at week 52 (aRR, 1.29; 95% CI, 0.99-1.67). The proportion of patients who achieved stability was lower among those with more severe airflow limitation (Figure 8). When analyzing a composite of no moderate/severe exacerbations, a ≤1-unit worsening in CAT, and no worsening in FEV_1_, a similar proportion of MT (19%) or PT patients (15%) achieved this composite vs the stability composite.

Using SGRQ as a sensitivity measure instead of CAT in the stability composite, a slightly higher proportion of patients achieved stability in the MT group vs when CAT was used (20% vs 18%, respectively; Figure 8). The treatment effect of MT was also higher among patients with chronic bronchitis (PT: 15%; MT: 20%) than those without chronic bronchitis (PT: 13%; MT: 14%) (Figure 8).

## Discussion

We have explored and characterized the endpoint of disease stability using post hoc analyses of 2 trials comparing inhaled treatments in patients with moderate-to-severe COPD and a pooled analysis of 3 trials of mepolizumab in COPD. Clinical trials often focus on group mean results for exacerbation rates or changes in lung function/patient-reported outcomes (PROs); however, stability describes the proportion of patients achieving a composite outcome, defined as no moderate/severe exacerbations and no worsening from baseline in CAT score and trough FEV_1_. Stability reflects a clinical state of low disease activity and can be regarded as a treatment target.^1^^,^^2^ Stability was achieved more often with triple therapy vs dual therapies in the IMPACT and FULFIL studies and with mepolizumab vs placebo in the MATINEE/METREX/METREO studies. Importantly, many patients who achieved stability in IMPACT also had improvements in CAT and FEV_1_, exceeding their MCIDs. Hence, achieving stability fulfills the GOLD treatment goal of no exacerbations^1^ while also reflecting clinically meaningful improvements in symptoms/quality of life. Additionally, the proportion of patients who achieved the individual stability components vs the composites in IMPACT highlights both the distinct nature of the components and a degree of interrelatedness, in line with existing evidence.^11^^,^^31^^,^^32^

Crucially, our data showed that achieving stability within 6 months was associated with reduced rates and risks of subsequent moderate/severe exacerbations and of ACM between 6 and 12 months. These results align with recent real-world evidence showing that achieving stability over 12 months can confer prognostic benefits on mortality and exacerbations for up to 10 years.^9^ Thus, aiming for stability in the short term may confer sustained benefits in the longer term.

The reduction in ACM risk was substantially greater when all 3 components were achieved than with DS-2 or exacerbation prevention alone. This highlights the importance of assessing all 3 components for stability, as each appears to contribute additively (perhaps even synergistically) to prognostic prediction. For practical reasons, spirometry is not routinely performed at every clinic visit, as reflected in the “DS-2” composite, which comprises exacerbations and CAT. While DS-2 may be a suitable surrogate that can be performed easily for practical reasons, it serves more as an indication of symptom burden than the more comprehensive 3-component stability composite.

At week 52 of IMPACT, a similar proportion of patients achieved no moderate/severe exacerbation, no worsening from baseline in CAT, or no worsening from baseline in FEV_1_ as individual endpoints (Figure 4), suggesting that no individual component was dominating the composite. At week 52, a greater proportion of exacerbation-free patients had no worsening from baseline in the other stability components, compared with patients who experienced exacerbations, suggesting a degree of relatedness between exacerbations and CAT/FEV_1_.

We observed that individuals who did not achieve stability at week 28 may nonetheless achieve stability at week 52. While COPD has been viewed as a progressive disease, the current results reinforce that the natural history of the disease is not necessarily linear in terms of progression.^33^ Furthermore, the ACM benefit after stability was achieved by week 28, suggesting potential value in maintaining periods of stability in this progressive disease.

Overall, our data show that the proportion of patients achieving stability was related to exacerbation history and (prior) use of pharmacotherapy in the trial populations: the highest proportion of patients achieved stability in the FULFIL study, followed by IMPACT and finally MATINEE/METREX/METREO. FULFIL did not enrich the trial population for exacerbation risk, in contrast to all the other studies. Additionally, MATINEE/METREX/METREO included patients with type 2 inflammation and persistent disease activity despite triple therapy. While caution is warranted when comparing populations across studies with different inclusion criteria, the higher exacerbation frequency and use of certain pharmacotherapies may indicate that patients in IMPACT and MATINEE/METREX/METREO had higher overall disease burden than in FULFIL.^19-22^ These results highlight that increased disease activity (particularly when reflected in exacerbations) or severity (reflected in airflow limitation) may reduce the capacity for patients to achieve stability. This has been further emphasized in populations with low exacerbation risk, in which up to 60% of patients achieved stability with UMEC/VI.^34^ Timely optimization of management, including after a single exacerbation occurs,^1^ could be crucial to prevent disease progression and the associated tissue damage.^4^^,^^32^

The treatment effect of FF/UMEC/VI vs UMEC/VI on stability at week 52 differed by blood eosinophils in IMPACT. This concurs with the now well-accepted ability of blood eosinophil counts to predict inhaled corticosteroid benefits in patients with increased exacerbation risk.^35^ The treatment effect of mepolizumab on stability in MATINEE/METREX/METREO was slightly higher in those with chronic bronchitis compared with those without.

Successful assessment and monitoring of stability over time rely on robust electronic medical records and regular recording of stability components.^1^ However, PROs and spirometry may not be routinely collected in primary care,^36^^,^^37^ and a lack of a standardized system for recording assessments can directly impact quality of care.^38-40^ Patient-reported outcomes, such as CAT (now called Chronic Airways Assessment Test [CAAT]), are easy and inexpensive to collect, and DS-2 (exacerbations and CAT) offers a pragmatic tool for primary care. We have also demonstrated that a more extensive PRO, such as the SGRQ, is a suitable measure of health status for use in a stability assessment.

This post hoc analysis has several strengths and limitations. Using randomized controlled trials as a data source provides a high level of robustness. This analysis also demonstrated stability in populations with varying degrees of exacerbation risk and prior pharmacotherapy usage; however, as these are post hoc analyses, further studies are needed to evaluate generalizability and examine stability in real-world settings and over extended follow-up. Additionally, it is important to highlight that the data used in this analysis relate to well-defined patient populations and use of specified triple inhaled, dual inhaled, or biologic therapies and thus are not reflective of broader therapy classes or patient populations. Furthermore, most of the stability components (FEV_1_, CAT/SGRQ) were assessed only at study visits. While this is a limitation of all stability analyses, it also reflects clinical practice, as disease progression will usually be assessed at scheduled visits or when key deteriorations occur (eg, exacerbations).

Future work could incorporate inflammatory and imaging biomarkers, as well as comorbidities, to expand the assessment of stability.^2^ The Bayesian joint modeling approach allowed us to analyze multiple outcomes while preserving the unique features of each variable and capturing their interdependencies. This approach provides robust uncertainty quantification through full posterior distributions and, if available, permits the incorporation of prior knowledge, which is especially beneficial when data are limited. Finally, as these analyses were primarily descriptive, they represent exploratory hypothesis generation and will require further, more robust statistical validation in future studies; in particular, further real-world assessment of stability as a treatment goal is needed to fully validate and support its implementation as a COPD treatment goal.

In conclusion, we characterized the composite endpoint of disease stability, defined as “no moderate/severe exacerbations from baseline and no worsening from baseline in CAT (or SGRQ) and FEV_1_ over 12 months,” across different pharmacological interventions, with each component of the composite contributing to a similar extent. Importantly, we have shown that stability is achievable in a meaningful proportion of patients and can be considered an attainable treatment target. Additionally, achieving stability even in the short term can confer longer-term clinical benefits in exacerbations and mortality, underscoring its potential clinical value as a treatment target. The results from the studies also showed how populations with different exacerbation histories and prior pharmacotherapy use may vary in the likelihood of achieving stability. While the 3-component stability composite is more comprehensive and confers the strongest prognostic benefit on mortality, we demonstrate the utility of DS-2 (exacerbations and CAT). Overall, implementing routine assessment of stability could enable optimal management that preserves lung function, reduces exacerbation risk, and improves long-term outcomes.

## Supplementary Material

aamag292_Supplementary_Data

## Data Availability

GSK makes available anonymized individual participant data and associated documents from interventional clinical studies that evaluate medicines, upon approval of proposals submitted to https://www.gsk-studyregister.com/en/. This article has an online data supplement, which is accessible from this issue’s table of contents online at www.atsjournals.org.
