# Supplementary material for: Defining disease stability in chronic obstructive pulmonary disease: evidence from phase 3 clinical trials
Source: Am J Respir Crit Care Med. 2026 Jun 11;212(9):2036–49. doi: 10.1093/ajrccm/aamag292 (PMC13519310; doi:10.1093/ajrccm/aamag292)
Supplement: Supplementary file 1 [file aamag292_supplementary_data.zip › aamag292_Supplementary_Data/aamag292 supp files.zip!/aamag292 supp files/COI_Disclosure_Form_MKH.docx]

**ICMJE DISCLOSURE FORM**

**Date: 21/01/2026**

**Your Name: MeiLan K. Han**

**Manuscript Title: Defining disease stability in COPD: Evidence from Phase 3 clinical trials**

**Manuscript number (if known): N/A**

**In the interest of transparency, we ask you to disclose all relationships/activities/interests listed below that are**

**related to the content of your manuscript. “Related” means any relation with for-profit or not-for-profit third**

**parties whose interests may be affected by the content of the manuscript. Disclosure represents a commitment**

**to transparency and does not necessarily indicate a bias. If you are in doubt about whether to list a relationship/activity/interest, it is preferable that you do so.**

**The following questions apply to the** **author’s relationships/activities/interests as they relate to the current**

**manuscript only.**

**The author’s relationships/activities/interests should be defined broadly. For example, if your manuscript pertains**

**to the epidemiology of hypertension, you should declare all relationships with manufacturers of antihypertensive medication, even if that medication is not mentioned in the manuscript.**

**In item #1 below, report all support for the work reported in this manuscript without time limit. For all other items,**

**the time frame for disclosure is the past 36 months.**

|  |  | **Name all entities with whom you have this relationship or indicate none (add rows as needed)** | | **Specifications/Comments**  **(e.g., if payments were made to you or to your institution)** |
| --- | --- | --- | --- | --- |
| **Time frame: Since the initial planning of the work** | | | | |
| 1 | All support for the present manuscript (e.g., funding, provision of study materials, medical writing, article processing charges, etc.)  **No time limit for this item.** | GSK | | Medical writing support, funded by GSK |
|  |  |  | | APC and Open access fees |
|  |  |  | |  |
|  |  |  | |  |
|  |  |  | |  |
|  |  |  | |  |
|  |  |  | |  |
|  |  |  | |  |
| **Time frame: past 36 months** | | | | |
| 2 | Grants or contracts from any entity (if not indicated in item #1 above). | NIH | In kind research support or funds paid to their institution | |
|  |  | Sanofi |  |  |
|  |  | Novartis |  |  |
|  |  | Nuvaira |  |  |
|  |  | Sunovion |  |  |
|  |  | Gala Therapeutics |  |  |
|  |  | COPD Foundation |  |  |
|  |  | AstraZeneca |  |  |
|  |  | American Lung Association |  |  |
|  |  | Boehringer Ingelheim |  |  |
|  |  | Biodesix |  |  |
| 3 | Royalties or licenses | UpToDate |  | |
|  |  | Norton Publishing |  | |
|  |  | Penguin Random House |  | |
| 4 | Consulting fees | AstraZeneca |  | |
|  |  | Boehringer Ingelheim |  | |
|  |  | GSK |  | |
|  |  | Novartis |  | |
|  |  | Pulmonx |  | |
|  |  | Teva |  | |
|  |  | Verona |  | |
|  |  | Merck |  | |
|  |  | Mylan |  | |
|  |  | Sanofi |  | |
|  |  | DevPro |  | |
|  |  | Aerogen |  | |
|  |  | Polarian |  | |
|  |  | Regeneron |  | |
|  |  | Altesa BioPharma |  | |
|  |  | Amgen |  | |
|  |  | Roche |  | |
|  |  | United Therapeutics |  | |
|  |  | RS Biotherapeutics |  | |
|  |  | Apreo Health |  | |
| 5 | Payment or honoraria for lectures, presentations, speakers bureaus, manuscript writing or educational events | Cipla | Payment or honoraria for consultancy | |
|  |  | Chiesi |  |  |
|  |  | AstraZeneca |  |  |
|  |  | Boehringer Ingelheim |  |  |
|  |  | GSK |  |  |
|  |  | Medscape |  |  |
|  |  | Integrity |  |  |
|  |  | NACE |  |  |
|  |  | Medwiz |  |  |
| 6 | Payment for expert testimony | ____None |  | |
|  |  |  |  | |
|  |  |  |  | |
| 7 | Support for attending meetings and/or travel | ____None |  | |
|  |  |  |  | |
|  |  |  |  | |
| 8 | Patents planned, issued or pending | ____None |  | |
|  |  |  |  | |
|  |  |  |  | |
| 9 | Participation on a Data  Safety Monitoring Board or Advisory Board | Novartis | Funds paid to institution | |
|  |  | Medtronic |  |  |
| 10 | Leadership or fiduciary role in other board, society, committee or advocacy group, paid or unpaid | COPD Foundation Board |  | |
|  |  | COPD Foundation Scientific Advisory Committee |  |  |
|  |  | American Thoracic Society (journal editor) |  | |
|  |  | ALA (volunteer spokesperson) |  | |
|  |  | GOLD scientific committee |  | |
|  |  | Emerson School Board, Ann Arbor, MI |  | |
| 11 | Stock or stock options | Meissa Vaccines |  | |
|  |  | Altesa BioPharma |  | |
|  |  |  |  | |
| 12 | Receipt of equipment, materials, drugs, medical writing, gifts or other services | ____None |  | |
|  |  |  |  | |
|  |  |  |  | |
| 13 | Other financial or non-financial interests | ____None |  | |
|  |  |  |  | |
|  |  |  |  | |

**Please place an “X” next to the following statement to indicate your agreement:**

**__X_ I certify that I have answered every question and have not altered the wording of any of the questions on this**

**form.**
