# Supplementary material for: Defining disease stability in chronic obstructive pulmonary disease: evidence from phase 3 clinical trials
Source: Am J Respir Crit Care Med. 2026 Jun 11;212(9):2036–49. doi: 10.1093/ajrccm/aamag292 (PMC13519310; doi:10.1093/ajrccm/aamag292)
Supplement: Supplementary file 1 [file aamag292_supplementary_data.zip › aamag292_Supplementary_Data/aamag292 supp files.zip!/aamag292 supp files/Defining_Disease_Stability_Supplement.docx]

**Online data supplement**

**Defining disease stability in COPD:
Evidence from Phase 3 clinical trials**

Dave Singh, Rianne Stacey, David MG Halpin, Surya P Bhatt, Jodie Crawford, Thomas Drury,

Valentina Di Boscio, Stefanie Kolterer, MeiLan K Han

**Supplementary methods: Studies**

The IMPACT and FULFIL trials have been described in detail previously(19, 20). IMPACT (NCT02164513) was a 52-week, Phase 3, randomized, double-blind, parallel-group, multicenter trial comparing once-daily single-inhaler triple therapy (SITT) with fluticasone furoate/umeclidinium/vilanterol (FF/UMEC/VI) 100/62.5/25 µg against once-daily dual therapy with either FF/VI 100/25 µg or UMEC/VI 62.5/25 µg, using 2:2:1 randomization(19). This post hoc analysis included the intention-to-treat (ITT) population of 10,355 patients; at screening 55% had ≥2 moderate/severe exacerbations in the prior 12 months, 64% had postbronchodilator FEV_1_, forced expiratory volume in 1 second (FEV₁) <50% predicted, and the mean (standard deviation [SD]) chronic obstructive pulmonary disease (COPD) assessment test (CAT) score was 20.1 (6.1). FULFIL (NCT02345161) was a 24-week, Phase 3, randomized, double-blind, double-dummy, parallel-group, multicenter trial comparing once-daily SITT with FF/UMEC/VI 100/62.5/25 µg against twice-daily dual therapy with budesonide/formoterol (BUD/FOR) 400/12 µg, using 1:1 randomization(20). This post hoc analysis included the ITT population of 1810 patients; at screening, though not enriched for exacerbations, 37% had ≥2 moderate/severe exacerbations in the prior 12 months, 54% had postbronchodilator FEV₁ <50% predicted, and the mean (SD) CAT score was 19.1 (5.2).

An additional analysis pooled the data from three pivotal Phase 3 studies in biologic therapy, the details of which have been described previously(21, 22). Briefly, MATINEE (NCT04133909), METREX (NCT02105948), and METREO (NCT02105948) were Phase 3, double-blind, randomized, placebo-controlled trials in patients continuing to exacerbate on triple therapy, comparing subcutaneous mepolizumab 100 mg versus placebo as an add on to a maintenance regimen of triple-inhaler therapy, administered every 4 weeks. The pooled population of these studies included 1146 patients with ≥2 moderate exacerbations or ≥1 severe exacerbations in the previous year, with blood eosinophils ≥300 cells/µL, who were classified between Global Initiative for Chronic Obstructive Lung Disease grades 2 and 4 and across the spectrum of the modified Medical Research Council dyspnea scale. Patients were enrolled regardless of the presence/absence of chronic bronchitis and/or emphysema. At screening, approximately 25% had ≥1 severe exacerbation in the prior 12 months; additionally, approximately 57% had postbronchodilator FEV₁ <50% predicted, and the mean (SD) CAT score was 19.2 (7.1).

**Supplementary methods: Statistical analysis**

*Prognostic value of the stability composite*

Analysis of the annualized rate of on-treatment moderate/severe exacerbations was performed using a generalized linear model assuming a negative binomial distribution and covariates of treatment group, sex, exacerbation history (≤1, ≥2 moderate/severe), smoking status (at screening), geographical region, postbronchodilator percent predicted FEV_1_ (at screening), and stability status at Week 28. Time to first moderate/severe exacerbation was analyzed using a Cox proportional hazards model with covariates of treatment group, sex (at screening), exacerbation history (≤1, ≥2 moderate/severe), smoking status (at screening), geographical region, postbronchodilator % predicted FEV_1_ (at screening), and stability status (at Week 28). For the all-cause mortality (ACM) analyses, probability of having event (prior to Day 365), 95% confidence interval (CI), first quartile and median time to onset were taken from the Kaplan–Meier estimates; hazard ratios (HR) and 95% CI were generated from a Cox proportional hazards model with covariates of treatment group, sex, age, and stability status at Week 28.

*Bayesian joint modeling*

To generate covariate-independent marginal posterior estimates, we used G-computation(41) by drawing a representative sample of 1000 patients from the overall dataset and combining their data with 2000 posterior samples of the model parameters. For each patient and posterior sample combination, we then simulated 100 outcomes via Gaussian copula values translated through normal and negative binomial quantile functions; we then assessed whether these outcomes met the no worsening thresholds and averaged the results across 1000 patients and 100 outcomes to estimate the marginal posterior distribution for the probability of achieving stability. This distribution was then summarized using the posterior mean and 95% highest posterior density credible intervals.

*Model specification*

For disease stability we have 3 component measures: change from baseline in trough FEV_1_ at Week 52, change from baseline in CAT score at Week 52, and number of moderate or severe exacerbations up to Week 52. For modeling purposes, we define these as $Y_{1}$, $Y_{2},$ and $Y_{3}$, respectively, assume they are distributed marginally as:

$$\begin{matrix} Y_{i1}\sim Normal\left( \mu_{i1}, \sigma_{i1} \right) & Y_{i2}\sim Normal\left( \mu_{i2}, \sigma_{i2} \right) & Y_{i3}\sim NegBin\left( r_{i}, \varphi_{i} \right) \end{matrix}$$

Where:

- $\mu_{i1}$ and$\sigma_{i1}$ are the normal mean and dispersion (SD) for $Y_{i1}$.
- $\mu_{i2}$ and$\sigma_{i2}$ are the normal mean and dispersion (SD) for $Y_{i2}$.
- $r_{i}$ and $\varphi_{i}$ are the negative binomial rate and dispersion for $Y_{i3}$.

We create three marginal models for each outcome and then combine these using a three-dimensional Gaussian copula $C_{123}\left[ \cdot,\cdot,\cdot\mid\boldsymbol{\theta} \right]$ to build a joint likelihood that allows each measure to be modeled simultaneously while allowing for dependence between the individual level outcomes through the matrix of copula dependence parameters $\boldsymbol{\theta}$.

We define models for the mean, variances, rates, dispersions, and copula dependence parameters as:

$$\left[ \begin{aligned} \mu_{i1} \\ \mu_{i2} \\ \log\left( r_{i} \right) \end{aligned} \right]=\left( \begin{aligned} &x_{i1}\alpha_{11}+x_{i2}\alpha_{12}+x_{i3}\alpha_{13}+\boldsymbol{X}_{i1}\boldsymbol{\beta}_{1} \\ &x_{i1}\alpha_{21}+x_{i2}\alpha_{22}+x_{i3}\alpha_{23}+\boldsymbol{X}_{i2}\boldsymbol{\beta}_{2} \\ &x_{i1}\alpha_{31}+x_{i2}\alpha_{32}+x_{i3}\alpha_{33}+\boldsymbol{X}_{i3}\boldsymbol{\beta}_{3} \end{aligned} \right),$$

$$\left[ \begin{matrix} \sigma_{i1} \\ \sigma_{i2} \\ \varphi_{i}^{-1} \end{matrix} \right]=\left( \begin{matrix} x_{i1}s_{11}+x_{i2}s_{12}+x_{i3}s_{13} \\ x_{i1}s_{21}+x_{i2}s_{22}+x_{i3}s_{23} \\ x_{i1}s_{31}+x_{i2}s_{32}+x_{i3}s_{33} \end{matrix} \right),\left[ \begin{aligned} \theta_{12} \\ \theta_{13} \\ \theta_{23} \end{aligned} \right]=\left( \begin{aligned} x_{i1}\omega_{1}^{12}+x_{i2}\omega_{2}^{12}+x_{i3}\omega_{3}^{12} \\ x_{i1}\omega_{1}^{13}+x_{i2}\omega_{2}^{13}+x_{i3}\omega_{3}^{13} \\ x_{i1}\omega_{1}^{23}+x_{i2}\omega_{2}^{23}+x_{i3}\omega_{3}^{23} \end{aligned} \right)$$

Where:

- $x_{it}$ is the treatment design matrix component for patient $i$, set up as indicator variables for treatment $t$.
- $\alpha_{jt}$ are the treatment effect parameters for the normal means and negative binomial rates.
- $\boldsymbol{X}_{ij}$ are vectors of remaining covariate design matrix components for patient $i$ and measure $j$.
- $\boldsymbol{\beta}_{j}$ are vectors of remaining covariate parameters for the normal means and negative binomial rates.
- $s_{jt}$ are the dispersion parameters of the $j$th measure for treatment $t$.
- $\omega_{t}^{jk}$ are the copula dependence parameters between the $j$-th and $k$-th measures for treatment $t$.

We then combine the above models using the corresponding cumulative distribution functions $F_{1}\left( Y_{i1}|\mu_{i1},\sigma_{i1} \right)$, $F_{2}\left( Y_{i2}|\mu_{i2},\sigma_{i2} \right)$, and $F_{3}\left( Y_{i3}|r_{i},\varphi_{i} \right)$ with the copula function $C_{123}\left[ \cdot,\cdot,\cdot\mid\boldsymbol{\theta} \right]$ to create a joint likelihood as a mixed data type density-mass function. The form of this likelihood is derived below. To simplify the notation in the following derivations, we drop the individual level index $i$ and the conditioning of the marginal model parameters on the cumulative distribution functions (CDF), i.e., $F_{1}\left( Y_{i1}|\mu_{i1},\sigma_{i1} \right)=F_{1}\left( Y_{1} \right)$, $F_{2}\left( Y_{i2}|\mu_{i2},\sigma_{i2} \right)=F_{2}\left( Y_{2} \right)$, and $F_{3}\left( Y_{i3}|r_{i},\varphi_{i} \right)=F_{3}\left( Y_{3} \right)$.

*Likelihood derivation*

Before starting to derive a likelihood we restate some results for multivariate distributions and copulas [1,2,3] that are used in the process. A general likelihood for a three-dimensional distribution with two continuous and one discrete margin is then derived for an arbitrary copula. Finally, properties and formulas for the chosen Gaussian copula and marginal distributions are used to simplify the likelihood to improve the performance for Bayesian modeling.

*Preliminaries*

A copula is a multivariate distribution function with uniform margins: i.e., for $n=1,\ldots,N$ dimensions.

$$\begin{matrix} C_{1:N}\left[ u_{1}, \ldots, u_{N}|\theta\right] & u_{1}, \ldots, u_{N} \sim uniform\left( 0,1 \right) \end{matrix}$$

These properties allow separate marginal univariate CDFs (uniformly distributed) to be embedded into a copula function to form a new joint multivariate distribution which retains the property of each margin but imposes a dependence relationship governed by the chosen copula, i.e.,

$$\begin{matrix} F_{1:N}\left( Y_{1}, \ldots, Y_{N} \right)=C_{1:N}\left[ F_{1}\left( Y_{1} \right), \ldots, F_{N}(Y_{N})|\boldsymbol{\theta} \right] & (1) \end{matrix}$$

Where:

- $F_{n}\left( Y_{n} \right)$ is CDF for the $n$-th dimension (variable).
- $\boldsymbol{\theta}$ are a set of copula dependence parameters.
- $C_{1:N}\left[ \cdot, \ldots, \cdot|\boldsymbol{\theta} \right]$ is the N-dimensional copula **distribution** function.
- $F_{1:N}\left( Y_{1}, \ldots, Y_{N} \right)$ is the new combined multivariate distribution function.

Dealing with joint CDFs is not very practical for model fitting and therefore the likelihood for the joint density-mass function is derived by taking the partial derivative of the continuous variables and performing finite differencing of the resulting function to get the discrete mass.

*General likelihood derivation*

In the case we are considering, we have two continuous margins and a single discrete margin. This means to derive the joint density-mass function to use as the likelihood essentially boils down to applying this high-level formula:

$\begin{matrix} f_{123}\left( Y_{1}, Y_{2},Y_{3}\mid\boldsymbol{\theta} \right)=\frac{{\partial^{2}C}_{123}\left[ F_{1}\left( Y_{1} \right), F_{2}\left( Y_{2} \right), F_{3}(Y_{3})|\boldsymbol{\theta} \right]}{\partial Y_{1}\partial Y_{2}}-\frac{{\partial^{2}C}_{123}\left[ F_{1}\left( Y_{1} \right), F_{2}\left( Y_{2} \right), F_{3}(Y_{3}-1)|\boldsymbol{\theta} \right]}{\partial Y_{1}\partial Y_{2}} & \left( 4 \right) \end{matrix}$

It can be shown using the chain rule of differentiation and results from *section 2.1.3 in Joe* [1] and *theorem 4.10 in Czado* [2] that the partial derivative of the joint density is made up of the joint density function for the differentiated margins and a conditional CDF for the remaining margins, which in our case is :

$$\frac{{\partial^{2}C}_{123}\left[ F_{1}\left( Y_{1} \right), F_{2}\left( Y_{2} \right), F_{3}(Y_{3})|\boldsymbol{\theta} \right]}{\partial Y_{1}\partial Y_{2}}=F_{3|21}(Y_{3}|Y_{2},Y_{1})\cdot c_{12}\left[ F_{1}\left( Y_{1} \right), F_{2}\left( Y_{2} \right)\mid\theta_{12} \right]\cdot f_{2}\left( Y_{2} \right)\cdot f_{1}\left( Y_{1} \right)$$

Where:

- $F_{3|21}(Y_{3}|Y_{2},Y_{1})$ is the conditional CDF of the discrete variable $Y_{3}$ given the values of the two continuous variables $Y_{1}$ and $Y_{2}$ but also the copula governing the relationship between the $Y_{1}$, $Y_{2}$, and $Y_{3}$ variables.
- $c_{12}\left[ F_{1}\left( Y_{1} \right), F_{2}\left( Y_{2} \right)\mid\theta_{12} \right]$ is a 2-dimensional copula **density** function for continuous variables $Y_{1}$ and $Y_{2}$.
- $f_{1}\left( Y_{1} \right)$ and $f_{2}\left( Y_{2} \right)$ are the univariate marginal PDFs for continuous variables $Y_{1}$ and $Y_{2}$.

Under the assumption that the distributional relationship between each pair of $Y_{1}$, $Y_{2}$ , and $Y_{3}$ does not depend on the other variable i.e. $F_{jk}\left( Y_{j},Y_{k} \right) \perp Y_{l}$ (generally referred to as “the simplifying assumption” for multivariate copula contructions) [3], it can be shown that the conditional CDF $F_{3|21}(Y_{3}|Y_{2},Y_{1})$ can be constructed from nested partial derivatives of bivariate copulas (sometimes called H-functions) specifically:

$\begin{matrix} F_{3|21}\left( Y_{3} | Y_{2},Y_{1} \right)=H_{3|21}^{C}\left[ F_{3|1}\left( Y_{3}\mid Y_{1} \right) | F_{2|1}\left( Y_{2}\mid Y_{1} \right), \theta_{23|1} \right]=\frac{\partial}{\partial F_{2|1}\left( Y_{2}\mid Y_{1} \right)}C_{23|1}\left[ F_{2|1}\left( Y_{2}\mid Y_{1} \right), F_{3|1}\left( Y_{3}\mid Y_{1} \right)|\theta_{23|1} \right] & \left( 5 \right) \end{matrix}$

$$\begin{matrix} F_{3|1}\left( Y_{3}\mid Y_{1} \right)=H_{3|1}^{C}\left[ F_{3}\left( Y_{3} \right) \mid F_{1}\left( Y_{1} \right), \theta_{13} \right]=\frac{\partial}{\partial F_{1}\left( Y_{1} \right)}C_{13}\left[ F_{1}\left( Y_{1} \right), F_{3}\left( Y_{3} \right)\mid\theta_{13} \right] & \left( 6 \right) \end{matrix}$$

$$\begin{matrix} F_{2|1}\left( Y_{2}\mid Y_{1} \right)=H_{2|1}^{C}\left[ F_{2}\left( Y_{2} \right)\mid F_{1}\left( Y_{1} \right), \theta_{12} \right]=\frac{\partial}{\partial F_{1}\left( Y_{1} \right)}C_{12}\left[ F_{1}\left( Y_{1} \right), F_{2}\left( Y_{2} \right)\mid\theta_{12} \right] & \left( 7 \right) \end{matrix}$$

Where $H^{C}\left[ \cdot\mid\cdot,\theta\right]$ are the bivariate h-functions (partial derivatives of bivariate copulas). This means we can build the joint density-mass function to use as the likelihood by first defining the marginal distributions, using the copula density for the continuous portions of the distribution, and then build up the conditional CDF using a series of partial derivatives of bivariate copulas. This leads the joint density:

$\begin{matrix} f_{123}\left( Y_{1}, Y_{2},Y_{3}\mid\boldsymbol{\theta} \right)=f_{1}\left( Y_{1} \right)\cdot f_{2}\left( Y_{2} \right)\cdot c_{12}\left[ F_{1}\left( Y_{1} \right), F_{2}\left( Y_{2} \right)\mid\theta_{12} \right]\cdot\left[ F_{3|21}\left( Y_{3} | Y_{2},Y_{1} \right)-F_{3|21}\left( Y_{3}-1 | Y_{2},Y_{1} \right) \right] & (8) \end{matrix}$

Where $F_{3|21}\left( Y_{3} | Y_{2},Y_{1} \right)$ and $F_{3|21}\left( Y_{3}-1 | Y_{2},Y_{1} \right)$ are formed in stages using $(7)$ and $(6)$ then combing into $(5)$. This is the generic joint distribution for two continuous and one discrete margin for an aribitary multivariate copula that satisfies the simplifying assumption. By definition, our choice of copula, the multivariate Gaussian copula, satisfies the simplifying assumption (see *theorem 5.2 in Czado* [2]) and therefore the structural formulation outline above can be applied. The H-functions and densities for each specific copula and can be looked up in the literature. The bivariate Gauss copula H-functions and density are:

$$\begin{matrix} F_{j|k}\left( Y_{j}|Y_{k} \right)=H_{j|k}^{Gauss}\left[ F_{j}\left( Y_{j} \right)|F_{k}\left( Y_{k} \right),\theta_{jk} \right]=\Phi\left[ \frac{\Phi^{-1}\left( F_{j}\left( Y_{j} \right) \right)-\theta_{jk}\cdot\Phi^{-1}\left( F_{k}\left( Y_{k} \right) \right)}{\sqrt{1-\theta_{jk}^{2}}} \right] & \left( 9 \right) \end{matrix}$$

$\begin{matrix} F_{j\mid kl}\left( Y_{j}|Y_{k}, Y_{l} \right)=H_{j|kl}^{Gauss}\left[ F_{j|l}\left( Y_{j}|Y_{l} \right) | F_{k|l}\left( Y_{k}|Y_{l} \right),\theta_{jk|l} \right]=\Phi\left[ \frac{\Phi^{-1}\left( F_{j|l}\left( Y_{j}|Y_{l} \right) \right)-\theta_{jk|l}\cdot\Phi^{-1}\left( F_{k|l}\left( Y_{k}|Y_{l} \right) \right)}{\sqrt{1-\theta_{jk|l}^{2}}} \right] & \left( 10 \right) \end{matrix}$

$$\begin{matrix} c_{jk}\left[ F_{j}\left( Y_{j} \right), F_{k}\left( Y_{k} \right)\mid\theta_{jk} \right]=\frac{\phi_{jk}\left( \Phi^{-1}\left[ F_{j}\left( Y_{j} \right) \right], \Phi^{-1}\left[ F_{k}\left( Y_{k} \right) \right]|\theta_{jk} \right)}{\phi_{j}\left( \Phi^{-1}\left[ F_{j}\left( Y_{j} \right) \right] \right)\cdot\phi_{k}\left( \Phi^{-1}\left[ F_{k}\left( Y_{k} \right) \right] \right)} & (11) \end{matrix}$$

Where $\phi$ and $\Phi$ are the Gaussian density and distribution functions respectively. Using (8) together with (9), (10) and (11) allows us to build the joint likelihood for two continuous and one discrete variable joined with a Gaussian copula. These formulas show all the constituent parts required to build the likelihood for the joint Bayesian model implemented.

*MCMC run and convergence*

The joint Bayesian model was fitted in SAS using PROC MCMC using its Metropolis-Hastings sampler with a custom likelihood specified via the GENERAL model statement.

The following priors were used for model parameters:

| **Parameter type** | **Prior distribution** |
| --- | --- |
| Marginal models for mean | $Normal\left( mean=0, SD=100 \right)$ |
| Marginal models for dispersion | $Inv.Gamma\left( shape=0.01, scale=0.01 \right)$ |
| Copula dependence | $Uniform(-1,1)$ |
|  |  |

After a “burn in” of 1000, a run of 60,000 samples with a thinning of 30 was completed giving a total of 2000 samples of the posterior distribution of model parameters. Posterior “worm plots” for each parameter with autocorrelation assessments were also produced and judged to be adequate.

*Posterior estimates of disease stability*

Following the MCMC run, each of the samples from the posterior were then merged to 1000 resampled patients from the total data in IMPACT (approximately 10,000). For each posterior sample and re-sampled patient, we then created posterior predictive means and variances using the individual patient covariate values. For each of these 200,000 posterior predictive means and variances, we jointly simulated 100 values using the Gaussian copula and corresponding univariate quantile functions (normal and negative binomial). Each set of simulations were then evaluated as above or below each set of thresholds being considered for disease stability. These 100 predictive values were then averaged over the 1000 re-sampled patients to obtain 2000 (G-computed) population based posterior estimates of the proportion of patients satisfying each set of potential thresholds. This posterior distribution was then summarized using the poster mean and SD together with 95% highest posterior difference credible intervals. Monte Carlo standard errors (MCSE) were calculated for each posterior mean using the posterior processing macros available from PROC MCMC.

The resampling of 1000 patients was due to the computational infeasibility of merging each of the 2000 posterior samples to the 10,000 IMPACT patients and then predicting 100 data values. Crunching through 2 billion rows of data was also thought to be unnecessary given the maximum MCSE for the proportions across all thresholds was 0.028 percent. This was further reinforced by running the model with five separate chains and calculating five separate estimates of the posterior mean of achieving the thresholds of no exacerbations and zero change from baselines in CAT and FEV_1_. The results indicated very little Monte Carlo variation and are summarized below:

| **Run** | **MCMC seed** | **Resample seed** | **Posterior mean of disease stability (%)** |
| --- | --- | --- | --- |
| 1 | 1111 | 11111 | 25.5763640 |
| 2 | 2222 | 22222 | 25.5682610 |
| 3 | 3333 | 33333 | 25.5537775 |
| 4 | 4444 | 44444 | 25.5573595 |
| 5 | 5555 | 55555 | 25.5419690 |

**References**

1. Joe H. Dependence Modeling with Copulas (1st ed.). Chapman and Hall/CRC; 2014.
2. Czado C. Analyzing Dependent Data with Vine Copulas: A Practical Guide with R. European Organization for Nuclear Research 2019.
3. Stöber J, Joe H, Czado C. Simplified pair copula constructions-Limitations and extensions. J Multivar Anal 2013; 119: 101-118.

**Supplementary Table E1. Baseline characteristics of patients in the FULFIL(****20****) and IMPACT(****19****) studies**

|  | **FULFIL (ITT population)** | | | **IMPACT (ITT population)** | | | |
| --- | --- | --- | --- | --- | --- | --- | --- |
|  | **FF/UMEC/VI 100/62.5/25 µg (n=911)** | **BUD/FOR 400/12 µg (n=899)** | **Total**  **(n=1810)** | **FF/UMEC/VI 100/62.5/25 µg (n=4151)** | **FF/VI  100/25 µg (n=4134)** | **UMEC/VI 62.5/25 µg (n=2070)** | **Total (n=10,355)** |
| Sex, n (%) |  |  |  |  |  |  |  |
| Male | 678 (74) | 663 (74) | 1341 (74) | 2766 (67) | 2748 (66) | 1356 (66) | 6870 (66) |
| Female | 233 (26) | 236 (26) | 469 (26) | 1385 (33) | 1386 (34) | 714 (34) | 3485 (34) |
| Age, mean (SD), years | 64.2 (8.6) | 63.7 (8.7) | 63.9 (8.6) | 65.3 (8.2) | 65.3 (8.3) | 65.2 (8.3) | 65.3 (8.3) |
| BMI, mean (SD), kg/m^2^ | 26.9 (5.2)* | 27.0 (5.1)^†^ | 26.9 (5.1)^‡^ | 26.6 (6.2)^§^ | 26.7 (6.1) | 26.6 (5.9) | 26.6 (6.1)^¶^ |
| Smoking status at screening, n (%) |  |  |  |  |  |  |  |
| Former smoker | 511 (56) | 505 (56) | 1016 (56) | 2715 (65) | 2711 (66) | 1342 (65) | 6768 (65) |
| Current smoker | 400 (44) | 394 (44) | 794 (44) | 1436 (35) | 1423 (34) | 728 (35) | 3587 (35) |
| Postbronchodilator FEV_1_, mean (SD), % predicted | 45.5 (13.0)** | 45.1 (13.6)^††^ | 45.3 (13.3)^‡‡^ | 45.7 (15.0)^§§^ | 45.5 (14.8)^¶¶^ | 45.4 (14.7)*** | 45.5 (14.8)^†††^ |
| Moderate/severe exacerbations in the previous year, n (%) |  |  |  |  |  |  |  |
| 0 | 313 (34) | 317 (35) | 630 (35) | 2 (<1) | 5 (<1) | 2 (<1) | 9 (<1) |
| 1 | 252 (28) | 253 (28) | 505 (28) | 1853 (45) | 1907 (46) | 931 (45) | 4691 (45) |
| ≥2 | 346 (38) | 329 (37) | 675 (37) | 2296 (55) | 2222 (54) | 1137 (55) | 5655 (55) |
| CAT score at screening, mean (SD) | 19.2 (5.2) | 19.1 (5.2) | 19.1 (5.2) | 20.1 (6.1) | 20.1 (6.1) | 20.2 (6.2) | 20.1 (6.1) |

*n=908; †n=897; ^‡^n=1805; ^§^n=4148; ^¶^n=10,352; **n=908; ^††^n=897; ^‡‡^n=1085; ^§§^n=4145; ^¶¶^n=4133; ***n=2069; ^†††^n=10,347.

BMI, body mass index; BUD, budesonide; CAT, COPD Assessment Test; COPD, chronic obstructive pulmonary disease; FEV_1_, forced expiratory volume in 1 second;
FF, fluticasone furoate; FOR, formoterol; ITT, intention-to-treat; n, number of patients; SD, standard deviation; UMEC, umeclidinium; VI, vilanterol

**Supplementary Table E2. Pooled baseline demographics of MATINEE/METREX/METREO(****21****,** **22****) and disease characteristics, stratified by GOLD grade***

|  | **GOLD 2^†^ (N=483)** | | **GOLD 3^†^ (N=504)** | | **GOLD 4^†^ (N=148)** | |
| --- | --- | --- | --- | --- | --- | --- |
|  | **Mepolizumab 100 mg SC (n=238)** | **Placebo (n=245)** | **Mepolizumab 100 mg SC (n=256)** | **Placebo (n=248)** | **Mepolizumab 100 mg SC (n=69)** | **Placebo (n=79)** |
| Age, years, mean (SD) | 66.2 (9.0) | 66.0 (8.3) | 66.2 (8.2) | 66.2 (8.3) | 65.2 (6.0) | 63.5 (6.9) |
| Female, n (%) | 82 (34) | 78 (32) | 88 (34) | 93 (38) | 18 (26) | 14 (18) |
| Smoking history, n (%) |  |  |  |  |  |  |
| Never smoked | 0 | 3 (1) | 1 (<1) | 4 (2) | 0 | 0 |
| Current smoker | 66 (28) | 65 (27) | 74 (29) | 72 (29) | 18 (26) | 23 (29) |
| Former smoker | 172 (72) | 177 (72) | 181 (71) | 172 (69) | 51 (74) | 56 (71) |
| Duration of COPD, years, median  (min–max) | 7.5 (1–39) | 7.0 (1–32) | 9.0 (1–40) | 8.0 (1–33) | 10.0 (2–45) | 10.0 (1–25) |
| Exacerbations in the 12 months prior to screening, n (%) |  |  |  |  |  |  |
| ≥1 moderate/severe exacerbation(s) | 238 (100) | 243 (>99) | 256 (100) | 247 (>99) | 69 (100) | 79 (100) |
| ≥1 moderate exacerbation(s) | 214 (90) | 218 (89) | 219 (86) | 214 (86) | 52 (75) | 62 (78) |
| ≥1 severe exacerbation(s) | 47 (20) | 44 (18) | 74 (29) | 65 (26) | 27 (39) | 28 (35) |
| ≥1 exacerbation(s) requiring ED visit but not hospitalization | 17 (7) | 32 (13) | 37 (14) | 29 (12) | 8 (12) | 7 (9) |
| ≥1 exacerbation(s) requiring hospitalization but not ICU | 44 (18) | 42 (17) | 72 (28) | 63 (25) | 25 (36) | 25 (32) |
| Cardiovascular comorbidity, n (%) |  |  |  |  |  |  |
| Yes | 55 (23) | 61 (25) | 64 (25) | 62 (25) | 22 (32) | 18 (23) |
| No | 183 (77) | 184 (75) | 192 (75) | 186 (75) | 47 (68) | 61 (77) |
| Other comorbidity, n (%) |  |  |  |  |  |  |
| Yes | 188 (79) | 206 (84) | 200 (78) | 193 (78) | 52 (75) | 59 (75) |
| No | 50 (21) | 39 (16) | 56 (22) | 55 (22) | 17 (25) | 20 (25) |
| mMRC score at screening^‡^, n (%) |  |  |  |  |  |  |
| <2 | 63 (26) | 75 (31) | 38 (15) | 43 (17) | 8 (12) | 10 (13) |
| ≥2 | 174 (73) | 170 (69) | 215 (84) | 205 (83) | 61 (88) | 68 (86) |
| Missing | 1 (<1) | 0 | 3 (1) | 0 | 0 | 1 (1) |
| Health-related quality of life, mean (SD) |  |  |  |  |  |  |
| Patients with assessment, n | 231 | 240 | 244 | 242 | 69 | 79 |
| SGRQ total score^§^ | 50.1 (17.7) | 48.8 (17.7) | 57.6 (16.6) | 56.5 (17.0) | 61.5 (16.8) | 61.9 (13.8) |
| Patients with assessment, n | 231 | 240 | 243 | 240 | 69 | 78 |
| CAT score^¶^ | 17.8 (7.0) | 17.9 (7.0) | 20.1 (7.1) | 19.8 (7.2) | 20.7 (7.1) | 21.1 (6.1) |
| Symptoms of chronic bronchitis**, n (%) |  |  |  |  |  |  |
| Patients with assessment, n | 232 | 240 | 245 | 243 | 69 | 79 |
| Yes | 160 (69) | 165 (69) | 166 (68) | 159 (65) | 48 (70) | 54 (68) |
| No | 72 (31) | 75 (31) | 79 (32) | 84 (35) | 21 (30) | 25 (32) |

*Percentages may not sum to 100 because of rounding. ^†^The severity of airflow obstruction was assessed based on the postbronchodilator FEV_1_ at screening according to the GOLD guidelines for COPD. A postbronchodilator FEV_1_ of 80% or more of the predicted normal value was considered to indicate mild airflow obstruction; 50% or more to less than 80%, moderate; 30% or more to less than 50%, severe; and less than 30%, very severe. FEV_1_ predicted values calculated using NHANES III reference equations. In total, 11 patients (5 mepolizumab/6 placebo) had mild airflow obstruction (GOLD grade 1 [mild]: FEV_1_ ≥80% predicted). ^‡^Grades on the mMRC dyspnea scale range from 0 to 4, with higher grades indi­cating more severe dyspnea. ^§^Total scores on the SGRQ range from 0 to 100, with higher scores indicating worse health status. ^¶^Scores on the CAT range from 0 to 40, with higher scores indicating greater disease effects. **Symptoms of chronic bronchitis were assessed based on responses at baseline to items on the SGRQ related to cough and sputum.

CAT, COPD Assessment Test; COPD, chronic obstructive pulmonary disease; ED, emergency department; FEV_1_, forced expiratory volume in 1 second; GOLD, Global Initiative for Chronic Obstructive Lung Disease; ICU, intensive care unit; mMRC, modified medical research council; N, number of patients by GOLD grade; n, number of patients by characteristic; NHANES, National Health and Nutrition Examination Survey; SC, subcutaneous; SD, standard deviation; SGRQ, St George’s Respiratory Questionnaire.

**Supplementary Table E3. Sensitivity analysis of primary IMPACT disease stability analysis using jump to reference multiple imputation**

|  | **FF/UMEC/VI 100/62.5/25 N=4151** | **FF/VI 100/25 N=4134** | **UMEC/VI 62.5/25 N=2070** |
| --- | --- | --- | --- |
| **FF/VI as reference** | | | |
| Stable at Week 52, n (%) | 996 (24) | 692 (17) | 385 (19) |
| aRR (95% CI) |  | 1.43 (1.30, 1.56) |  |
| **UMEC/VI as reference** | | | |
| Stable at Week 52, n (%) | 1009 (24) | 709 (17) | 393 (19) |
| aRR (95% CI) |  |  | 1.29 (1.16, 1.43) |

aRR, adjusted risk ratio; CI, confidence interval; FF, fluticasone furoate; N, number of patients by category; n, number of patients who were stable; UMEC, umeclidinium; VI, vilanterol.

**Supplementary Table E4. Stability status at Week 52 by treatment split by baseline blood eosinophils and smoking status at screening**

|  | **FF/UMEC/VI 100/62.5/25 N=4151** | **FF/VI 100/25 N=4134** | **UMEC/VI 62.5/25 N=2070** |
| --- | --- | --- | --- |
| **Baseline blood eosinophils** |  |  |  |
| *<100* cells/μL |  |  |  |
| N | 1065 | 1014 | 512 |
| Stable at Week 52, n (%) | 213 (20) | 150 (15) | 92 (18) |
| Not stable at Week 52, n (%) | 852 (80) | 864 (85) | 420 (82) |
| *≥100–<300* cells/μL |  |  |  |
| N | 2215 | 2198 | 1085 |
| Stable at Week 52, n (%) | 496 (22) | 270 (12) | 175 (16) |
| Not stable at Week 52, n (%) | 1719 (78) | 1928 (88) | 910 (84) |
| *≥300* cells/μL |  |  |  |
| N | 863 | 913 | 468 |
| Stable at Week 52, n (%) | 184 (21) | 157 (17) | 62 (13) |
| Not stable at Week 52, n (%) | 679 (79) | 756 (83) | 406 (87) |
| **Smoking status at screening** |  |  |  |
| *Current smoker* |  |  |  |
| N | 1436 | 1423 | 728 |
| Stable at Week 52, n (%) | 299 (21) | 193 (14) | 123 (17) |
| Not stable at Week 52, n (%) | 1137 (79) | 1230 (86) | 605 (83) |
| *Former smoker* |  |  |  |
| N | 2715 | 2711 | 1342 |
| Stable at Week 52, n (%) | 597 (22) | 385 (14) | 207 (15) |
| Not stable at Week 52, n (%) | 2118 (78) | 2326 (86) | 1135 (85) |

Note: Patients who have missing CAT responses or FEV_1_ measurements will be considered as the

Disease stability criteria not being met for that timepoint. Includes on-treatment data only.

FF, fluticasone furoate; N, number of patients by category; n, number of patients per characteristic; UMEC, umeclidinium; VI, vilanterol.

**Supplementary Table E5. Proportion of patients achieving individual components at Week 28 in IMPACT**

|  | **IMPACT (Week 28, ITT)** | | |
| --- | --- | --- | --- |
|  | **FF/UMEC/VI 100/62.5/25 µg N=4151** | **FF/VI 100/25 µg**  **N=4134** | **UMEC/VI 62.5/25 µg**  **N=2070** |
| **Patients achieving the endpoint, n (%)** |  |  |  |
| CAT score |  |  |  |
| ≥0-unit *improvement* from baseline | 2346 (57) | 2059 (50) | 1024 (49) |
| ≥2-unit *improvement* from baseline | 1844 (44) | 1594 (39) | 789 (38) |
| Trough FEV_1_ |  |  |  |
| ≥0 mL *improvement* from baseline | 2567 (62) | 1701 (41) | 995 (48) |
| ≥100 mL *improvement* from baseline | 1740 (42) | 983 (24) | 603 (29) |

Note: Patients did not have an “improvement” status derived if baseline trough FEV_1_/CAT score was missing and at a particular visit where the trough FEV_1_ /CAT score was missing but subsequent on-treatment trough FEV_1_ assessments were present. Includes on-treatment data only.

CAT, COPD Assessment Test; COPD, chronic obstructive pulmonary disease; FEV_1_, forced expiratory volume in 1 second; FF, fluticasone furoate; ITT, intention-to-treat; UMEC, umeclidinium; VI, vilanterol.

**Supplementary Table E6. Proportion of patients achieving varying SGRQ thresholds at Week 28 and Week 52 in IMPACT**

|  | **IMPACT (Week 28, ITT population)** | | | **IMPACT (Week 52, ITT population)** | | |
| --- | --- | --- | --- | --- | --- | --- |
|  | **FF/UMEC/VI 100/62.5/25 µg N=4151** | **FF/VI**  **100/25 µg**  **N=4134** | **UMEC/VI 62.5/25 µg**  **N=2070** | **FF/UMEC/VI 100/62.5/25 µg N=4151** | **FF/VI**  **100/25 µg**  **N=4134** | **UMEC/VI**  **62.5/25 µg**  **N=2070** |
| **Patients achieving the endpoint, n (%)** | | | |  |  |  |
| ≤0-unit change from baseline in SGRQ total score | 2406 (59) | 1958 (48) | 966 (47) | 2203 (54) | 1181 (44) | 912 (44) |
| ≥4-unit *improvement* from baseline in SGRQ total score | 1870 (46) | 1492 (37) | 736 (36) | 1723 (42) | 1390 (34) | 696 (34) |
| ≤4-unit *worsening* from baseline in SGRQ total score | 2729 (68) | 2392 (62) | 1201 (62) | 2490 (62) | 2169 (56) | 1089 (56) |

Note: Patients did not have a “worsening” or “improvement” status derived if baseline trough FEV_1_/CAT score was missing and at a particular visit where the trough
FEV_1_/CAT score was missing but subsequent on-treatment trough FEV_1_ assessments were present. Includes on-treatment data only.

CAT, COPD Assessment Test; COPD, chronic obstructive pulmonary disease; FEV_1_, forced expiratory volume in 1 second; FF, fluticasone furoate; ITT, intention-to-treat; SGRQ, St George’s Respiratory Questionnaire; UMEC, umeclidinium; VI, vilanterol.

**Supplementary Table E7. Proportion of patients achieving various thresholds for CAT score and trough FEV_1_ at Week 28 or 52 in IMPACT, stratified by exacerbation status at Week 52**

| **Patients who achieved the endpoint, n (%)** | **FF/UMEC/VI**  **(N=4151)** | **FF/VI**  **(N=4134)** | **UMEC/VI**  **(N=2070)** | **FF/UMEC/VI**  **(N=4151)** | **FF/VI**  **(N=4134)** | **UMEC/VI**  **(N=2070)** |
| --- | --- | --- | --- | --- | --- | --- |
|  | **Exacerbation-free up to Week 52** | | | **Experienced a moderate or severe exacerbation** | | |
|  | n=1964 | n=1815 | n=904 | n=2187 | n=2319 | n=1166 |
| **CAT score (Week 28 vs baseline)** | | | | | | |
| ≥4-unit *improvement* | 726 (37) | 620 (34) | 327 (36) | 608 (28) | 536 (23) | 254 (22) |
| 3-unit *improvement* | 135 (7) | 113 (6) | 49 (5) | 106 (5) | 115 (5) | 51 (4) |
| 2-unit *improvement* | 129 (7) | 108 (6) | 53 (6) | 140 (6) | 102 (4) | 55 (5) |
| 1-unit *improvement* | 145 (7) | 103 (6) | 56 (6) | 122 (6) | 121 (5) | 57 (5) |
| No change | 123 (6) | 110 (6) | 64 (7) | 112 (5) | 131 (6) | 58 (5) |
| 1-unit *worsening* | 104 (5) | 86 (5) | 47 (5) | 142 (6) | 121 (5) | 52 (4) |
| 2-unit *worsening* | 90 (5) | 76 (4) | 44 (5) | 96 (4) | 104 (4) | 51 (4) |
| 3-unit *worsening* | 79 (4) | 96 (5) | 31 (3) | 90 (4) | 77 (3) | 55 (5) |
| ≥4-unit *worsening* | 236 (12) | 250 (14) | 120 (13) | 316 (14) | 335 (14) | 143 (12) |
| **Trough FEV_1_ (Week 28 vs baseline)** | | | | | | |
| ≥100 mL *improvement* | 982 (50) | 542 (30) | 327 (36) | 758 (35) | 441 (19) | 276 (24) |
| >75 mL–<100 mL *improvement* | 98 (5) | 94 (5) | 52 (6) | 96 (4) | 84 (4) | 53 (5) |
| >50 mL–<75 mL *improvement* | 109 (6) | 92 (5) | 49 (5) | 98 (4) | 87 (4) | 50 (4) |
| >25 mL–<50 mL *improvement* | 83 (4) | 75 (4) | 46 (5) | 107 (5) | 84 (4) | 39 (3) |
| >0 mL–<25 mL *improvement* | 101 (5) | 99 (5) | 43 (5) | 135 (6) | 103 (4) | 60 (5) |
| >0 mL–<25 mL *worsening* | 61 (3) | 59 (3) | 42 (5) | 78 (4) | 78 (3) | 28 (2) |
| >25 mL–<50 mL *worsening* | 62 (3) | 66 (4) | 30 (3) | 80 (4) | 95 (4) | 43 (4) |
| >50 mL–<75 mL *worsening* | 62 (3) | 75 (4) | 43 (5) | 86 (4) | 106 (5) | 35 (3) |
| >75 mL–<100 mL *worsening* | 68 (3) | 90 (5) | 31 (3) | 58 (3) | 79 (3) | 45 (4) |
| ≥100 mL *worsening* | 201 (10) | 414 (23) | 154 (17) | 286 (13) | 559 (24) | 178 (15) |
| **CAT score (Week 52 vs baseline)** | | | | | | |
| ≥4-unit *improvement* | 729 (37) | 581 (32) | 309 (34) | 534 (24) | 488 (21) | 237 (20) |
| 3-unit *improvement* | 124 (6) | 108 (6) | 41 (5) | 96 (4) | 83 (4) | 38 (3) |
| 2-unit *improvement* | 106 (5) | 128 (7) | 59 (7) | 109 (5) | 103 (4) | 46 (4) |
| 1-unit *improvement* | 110 (6) | 106 (6) | 51 (6) | 100 (5) | 100 (4) | 38 (3) |
| No change | 114 (6) | 88 (5) | 54 (6) | 115 (5) | 90 (4) | 51 (4) |
| 1-unit *worsening* | 119 (6) | 106 (6) | 54 (6) | 109 (5) | 91 (4) | 42 (4) |
| 2-unit *worsening* | 97 (5) | 79 (4) | 38 (4) | 86 (4) | 76 (3) | 36 (3) |
| 3-unit *worsening* | 83 (4) | 76 (4) | 34 (4) | 77 (4) | 83 (4) | 36 (3) |
| ≥4-unit *worsening* | 252 (13) | 258 (14) | 137 (15) | 310 (14) | 335 (14) | 150 (13) |
| **Trough FEV_1_ (Week 52 vs baseline)** | | | | | | |
| ≥100 mL *improvement* | 911 (46) | 519 (29) | 316 (35) | 620 (28) | 379 (16) | 232 (20) |
| >75 mL–<100 mL *improvement* | 108 (5) | 77 (4) | 27 (3) | 76 (3) | 58 (3) | 36 (3) |
| >50 mL–<75 mL *improvement* | 102 (5) | 81 (4) | 36 (4) | 89 (4) | 65 (3) | 24 (2) |
| >25 mL–<50 mL *improvement* | 89 (5) | 67 (4) | 44 (5) | 87 (4) | 66 (3) | 25 (2) |
| >0 mL–<25 mL *improvement* | 121 (6) | 101 (6) | 51 (6) | 98 (4) | 92 (4) | 45 (4) |
| >0 mL–<25 mL *worsening* | 58 (3) | 64 (4) | 36 (4) | 85 (4) | 57 (2) | 31 (3) |
| >25 mL–<50 mL *worsening* | 59 (3) | 71 (4) | 40 (4) | 69 (3) | 86 (4) | 35 (3) |
| >50 mL–<75 mL *worsening* | 64 (3) | 81 (4) | 38 (4) | 84 (4) | 84 (4) | 37 (3) |
| >75 mL–<100 mL *worsening* | 56 (3) | 98 (5) | 45 (5) | 73 (3) | 88 (4) | 38 (3) |
| ≥100 mL *worsening* | 235 (12) | 411 (23) | 167 (18) | 282 (13) | 515 (22) | 187 (16) |

Note: data presented only for patients with CAT score or FEV_1_ measurements available at Week 52. Patients did not have a “improvement” or “worsening” status derived if baseline trough FEV_1_/CAT score was missing and at a particular visit where the trough FEV_1_ /CAT score was missing but subsequent on-treatment trough FEV_1_ assessments were present. Patients who discontinued study prior to the timepoint of interest will not be considered to have met the 'No moderate/severe exacerbations' criteria. Includes on-treatment data only.

CAT, COPD Assessment Test; COPD, chronic obstructive pulmonary disease; FEV_1_, forced expiratory volume in 1 second; FF, fluticasone furoate; N, number of patients per treatment group; n, number of patients per characteristic; UMEC, umeclidinium; VI, vilanterol.

**Supplementary Table E8. Analysis of time to first moderate/severe exacerbations and time to ACM after Week 28 by stability status at Week 28**

|  | **Stability status at Week 28** | | | |
| --- | --- | --- | --- | --- |
|  | **Stable N=2456** | **Not stable (all) N=7899** | **Not stable (due to exacerbation*) N=3496** | **Not stable (not due to exacerbation^†^) N=4403** |
| **Including off-treatment data (with Additional Vital Status follow-up)** | | | | |
| *Time to ACM after Week 28* | | | | |
| N | 2446 | 6173 | 3006 | 3167 |
| Number of subjects with an event, n (%) | 16 (<1) | 84 (1) | 34 (1) | 50 (2) |
| Hazard ratio (95% CI) |  | 0.48 (0.28, 0.83) | 0.57 (0.31, 1.03) | 0.42 (0.24, 0.75) |
| Percent reduction in risk (95% CI) |  | 51.7 (17.4, 71.8) | 43.0 (-3.5, 68.6) | 57.7 (25.5, 75.9) |
| **On-treatment only** | | | | |
| *Time to first moderate/severe exacerbation after Week 28* | | | | |
| N | 2446 | 6150 | 2988 | 3162 |
| Number of subjects with an event, n (%) | 451 (18) | 1997 (32) | 1313 (44) | 684 (22) |
| Hazard ratio (95% CI) |  | 0.54 (0.49, 0.60) | 0.38 (0.34, 0.42) | 0.84 (0.75, 0.95) |
| Percent reduction in risk (95% CI) |  | 45.7 (39.8, 51.0) | 62.0 (57.7, 65.9) | 15.7 (5.0, 25.2) |

*May include patients who also failed on CAT and/or FEV_1_. ^†^Failed CAT and/or FEV_1_ (but did not fail exacerbation).

Note: Hazard ratio and 95% CI were generated using a Cox proportional hazards model with covariates of treatment group, sex, age, and disease stability status at Week 28. Percentage reduction in risk is calculated as (1-hazard ratio)*100.

ACM, all-cause mortality; CAT, COPD Assessment Test; CI, confidence interval; COPD, chronic obstructive pulmonary disease; FEV_1_, forced expiratory volume in 1 second; N, number of subjects; n, number of subjects with an event.

**Supplementary Table E9. Annualized rate of on-treatment moderate/severe COPD exacerbations after Week 28 by stability status at Week 28**

|  | **Stability status at Week 28** | | | |
| --- | --- | --- | --- | --- |
|  | **Stable N=2456** | **Not stable (all) N=7899** | **Not stable  (due to exacerbation*) N=3496** | **Not stable  (not due to exacerbation^†^) N=4403** |
| N | 2445 | 6144 | 2988 | 3156 |
| Model estimated annual exacerbation rate (95% CI) | 0.50  (0.46, 0.55) | 0.94 (0.90, 0.98) | 1.32  (1.25, 1.39) | 0.58  (0.54, 0.63) |
| Rate ratio (95% CI) |  | 0.53  (0.48, 0.59) | 0.38  (0.35, 0.42) | 0.86  (0.77, 0.97) |
| Percent reduction in annual rate (95% CI) |  | 47  (41, 52) | 62  (58, 65) | 14  (3, 23) |

*May include patients who also failed on CAT and/or FEV_1_. ^†^Failed CAT and/or FEV_1_ (but did not fail exacerbation).
Note: Analysis performed using a generalized linear model assuming a negative binomial distribution and covariates of treatment group, sex, exacerbation history (<=1, >=2 moderate/severe), smoking status (Screening), geographical region, post-bronchodilator percent predicted FEV_1_ (Screening) and disease stability status at Week 28.

CAT, COPD Assessment Test; CI, confidence interval; COPD, chronic obstructive pulmonary disease; FEV_1_, forced expiratory volume in 1 second; N, number of patients.

**Supplementary Figure E1. Kaplan–Meier plots of time to ACM after Week 28 (on- and off- treatment with Additional Vital Status follow-up) by stability status at Week 28, according to whether patients did or did not have an exacerbation**

*May have also not met CAT and/or FEV_1_; ^†^failed CAT and/or FEV_1_ (but did not fail exacerbation).

ACM, all-cause mortality; CAT, COPD Assessment Test; CI, confidence interval COPD, chronic obstructive pulmonary disease; FEV_1_, forced expiratory volume in 1 second; HR, hazard ratio.

**Supplementary Figure E2. Posterior probability of patients receiving (A) FF/VI or (B) UMEC/VI achieving no moderate/severe exacerbations, CAT and trough FEV_1_ thresholds using Bayesian joint modeling via Copula Marginal Regression**

Note: DS at Week 52 was defined as no moderate/severe exacerbations since baseline, change from baseline in CAT total score of ≤X and change from baseline in trough FEV_1_ ≥Y (mL); posterior probability of DS was estimated jointly using Bayesian Gauss copula marginal regression. All marginal models included terms for treatment, smoking status at screening, and geographical region; the marginal models for change from baseline in trough FEV_1_ and CAT total score included corresponding terms for baseline; the marginal exacerbation model also included terms for sex and exacerbation history. The contour surface plot shows the posterior probability of achieving no moderate/severe exacerbations together achieving combinations of change from baseline in CAT total score (x-axis) and change from baseline in trough FEV_1_ (y-axis). As an example – achieving no exacerbations, least a zero change from baseline in CAT score, and at least a zero change in trough FEV_1_ would be the contour values at the point [0,0] on the plot.

CAT, COPD Assessment Test; COPD, chronic obstructive pulmonary disease; DS, disease stability; FEV_1_, forced expiratory volume in 1 second; FF, fluticasone furoate; UMEC, umeclidinium; VI, vilanterol.
